# Predicted Cohesive Properties of a TATB Polymer-Bonded Insensitive High Explosive

**DOI:** 10.1021/acs.jpcc.6c03771

**Published:** 2026-10-07

**Authors:** Leo W. Zella, Nicholas T. Liesen, H. Keo Springer, Matthew P. Kroonblawd

**Affiliations:** Physical and Life Sciences Directorate, 4578 Lawrence Livermore National Laboratory, Livermore, California 94550, United States

## Abstract

Polymer-bonded explosives (PBXs) are composite materials whose structure–property relationships are poorly understood due to their complex microstructure. Microstructural adhesion between the energetic molecular crystals and the polymer binder in a PBX affects key properties including mechanical strength, safety, and aging characteristics, but is difficult to measure experimentally. To this end, we develop a molecular dynamics (MD) modeling framework to predict the cohesive properties of a prototypical PBX based on the insensitive high explosive TATB (1,3,5-triamino-2,4,6-trinitrobenzene) and a fluoropolymer binder. The MD framework rests on two key capabilities: (1) general protocols to construct MD simulations of crystal-polymer and crystal–crystal interfaces in low-symmetry materials, and (2) a numerically robust approach to compute the surface and interface energies. Formal analysis of the surface energy equation shows that its direct evaluation is poorly conditioned for systems involving polymers. In this respect, we adopt a regression-based approach for computing surface energies and extend it to interfaces. Application to idealized, defect-free TATB facets interfaced with a fluoropolymer reveals several energetic drivers, including a thermodynamic driving force for grain coarsening and a greater propensity for TATB crystallites to adhere to each other than to the polymer binder. These data indicate that the thermodynamic landscape governing formulation design and aging of PBXs is complex and involves additional variables besides crystal-polymer adhesion. While the present study focuses on explosives, the MD methodology is general and can be readily adapted to model other material systems involving low-symmetry crystals, including organic electronics, pharmaceuticals, and advanced polymer composites.

## Graphical abstract

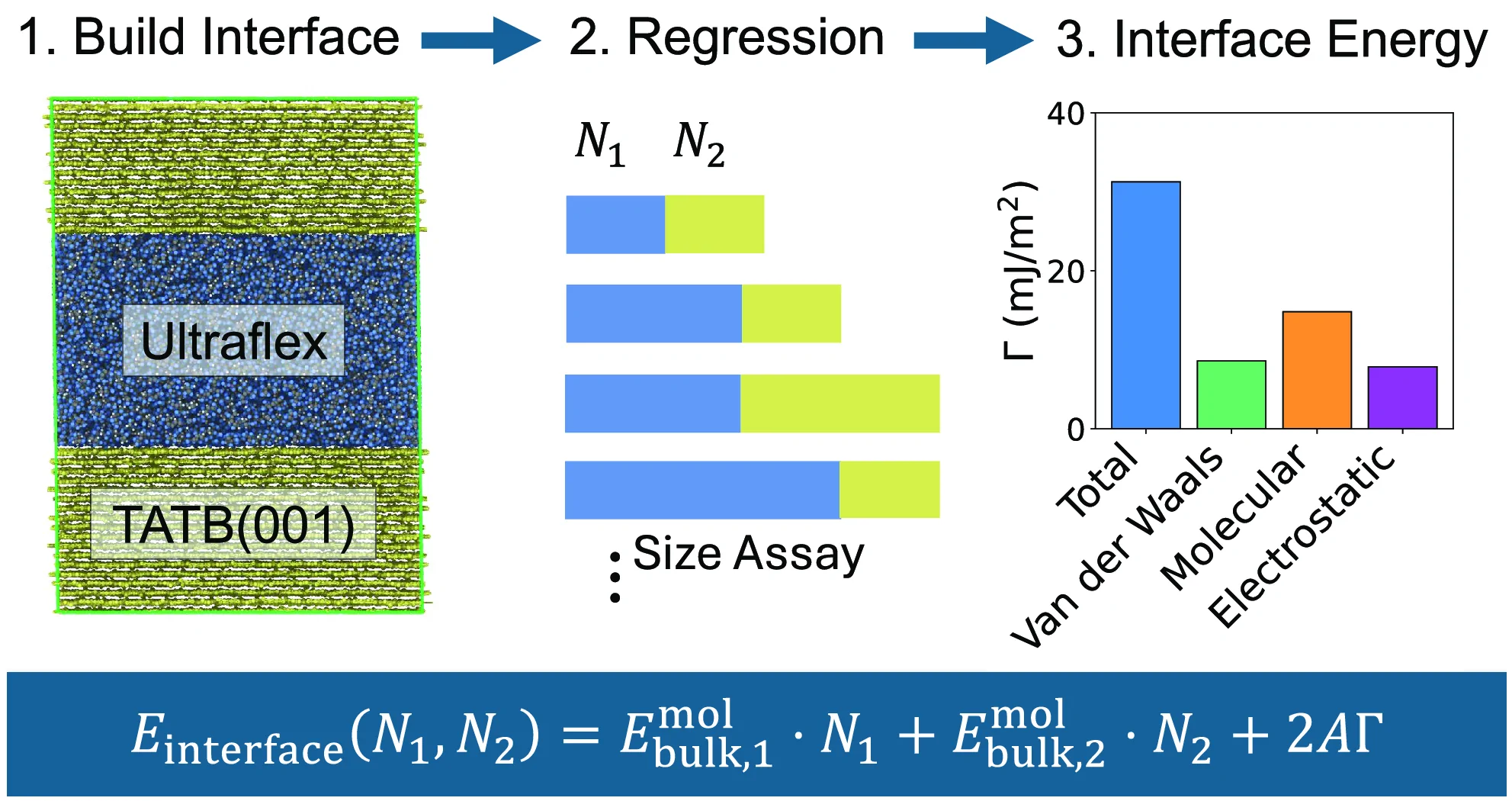

## 1 Introduction

Many common polymer-bonded explosives (PBXs) are composite materials consisting of energetic organic molecular crystals bound together by a polymeric binder, typically 5 to 10 wt %,^1^ often with binder-rich interphases that partially coat crystal surfaces. During the formation of a PBX part, a compaction step is commonly applied, and inadequate compaction results in PBXs that are poorly formed or defect ridden.^2^ Following successful compaction, PBXs must maintain mechanical integrity which includes resistance to creep^3,4^ and resilience to cracking and other failure modes under compression, tension, and thermal cycling.^4,5^ Under thermomechanical loading, PBXs are thought to accumulate damage through debonding at the crystal-binder interface, which can create voids and promote crack growth.^4,6–10^ Improving adhesion between the crystallite and the polymeric binder can improve the mechanical properties of PBXs,^10–13^ and potentially mitigate aging phenomena such as ratchet growth,^14,15^ but identifying an ideal polymeric binder for a given energetic crystal requires design principles rooted in the structure–property relationships that govern adhesion. Structure–property relationships for crystal-polymer adhesion are not well understood, and uncovering these relationships could inform PBX formulation design.^14^ A major challenge is that PBX microstructures are strongly heterogeneous, evolve with time due to aging, and have multiple relevant length scales, with void diameters spanning nanometers to micrometers^8,16^ and a variety of crystal facets expressed with grain size distributions spanning submicron to millimeter scales,^16–18^ which leads to inhomogeneities on the scale of millimeters or more.^5,16,18^ Isolating the specific role that crystal-binder adhesion plays in macroscopic mechanical response is difficult, but holds promise to improve the design of PBXs with enhanced toughness and resistance to creep, ratchet growth, and damage evolution.

Adhesion energy is the amount of thermodynamic work required to separate the two phases of a composite material per unit of interfacial area. While adhesion can be probed through measurements such as pull-off forces using atomic force microscopy (AFM),^19^ direct quantitative measurements of adhesion between two candidate PBX constituents are experimentally difficult due to the complex microstructure and small size of high-quality energetic crystals.^14^ As a result, the work of adhesion is often estimated indirectly from the surface energies of the two constituent materials.^14^ Surface energies can be measured via contact angle measurements^20^ and related techniques such as the Wilhelmy Plate method,^21^ or from other techniques such as inverse gas chromatography (IGC).^22^ With these techniques, the surface energy γ is calculated as the sum of the polar (γ_p_) and dispersive (γ_d_) components and the adhesion energy is inferred using a mixing rule.^14,23^

All-atom molecular dynamics (MD) simulations can directly calculate the energy required to form idealized crystal-polymer interfaces and simplify the attribution of the interfacial energies and surface energies to structure by direct control over the morphology.^24^ The standard MD approach to compute surface energies of liquids is via the so-called mechanical approach, which relates the surface tension to a spatial integral over the stress tensor using the Kirkwood-Buff equation.^25^ An analogous mechanical expression can be used to compute the free energy of liquid–liquid interfaces.^26^ However, these expressions cannot be accurately applied to interfaces with solids because creating a new solid surface or solid-polymer interface will include additional elastic and structural contributions (for example, surface stress contributions).^26^ Alternative free-energy methods exist for computing the surface and interface free energy that can be applied to PBXs,^27–29^ but these treatments are highly involved. In the case of surface energy, MD simulations of multiple energetic crystals have demonstrated the internal energy contribution significantly outweighs the entropic component for molecularly smooth surfaces.^29^ Even with a reliable estimate of the surface/interface free energy, an auxiliary calculation that isolates the contribution due to internal energy is also desirable for elucidating the impact of various physical and chemical factors that govern adhesion.

A standard way to calculate the surface energy of a solid through MD is to simulate two separate systems, with one containing free surfaces exposed to vacuum and the other corresponding to a 3D periodic bulk. From these two systems, one can compute the surface energy as

1 $$\gamma =\frac{E_{\mathrm{surf}}-E_{\mathrm{bulk}}}{2A}$$

Here, *E*_bulk_ is the potential energy of a 3D-periodic system with *N*_mol_ molecules at equilibrium, *E*_surf_ is the potential energy of a 2D thin film with *N*_mol_ molecules at equilibrium that is produced by introducing vacuum along a single cell direction, and *A* is the cross-sectional area of the surface exposed to the vacuum. The factor of 2 arises due to the formation of two free surfaces given periodic boundary conditions in the MD simulations. An analogous expression holds for the interface energy and it should be noted that this expression yields the surface energy without entropic contribution. While evaluating eq 1 appears straightforward, it suffers from poor numerical conditioning and small uncertainties in the calculated bulk energy can propagate to yield statistically ambiguous results.^30^ Recasting the surface energy calculation as a linear regression problem improves stability^31–34^ and has been independently “discovered” multiple times,^35^ but has not seemingly been applied to interface energy calculations.

In this work, we extend the surface energy regression approach and assemble an MD simulation construction toolkit based on the generalized crystal-cutting method^36^ (GCCM) to predict the energetics of crystal-polymer and crystal–crystal interfaces in a representative PBX. Our model system is comprised of the insensitive high explosive (HE) TATB (1,3,5-triamino-2,4,6-trinitrobenzene) and the fluoropolymer binder Ultraflex (see Figure 1). TATB is a layered triclinic molecular crystal^37^ that exhibits what is arguably the greatest anisotropy in its thermomechanical properties of any organic explosive in common use. Established TATB-based PBX formulations use fluoropolymer binders,^38,39^ and we focus on Ultraflex, which is a 70:30 random linear copolymer comprised of vinylidene fluoride (VDF) and hexafluoropropylene (HFP) which closely matches the reported experimental comonomer ratio.^40^ While TATB is a technologically relevant material in its own right,^38,39^ we focus on it as a case study here for two key reasons. First, because TATB crystal is triclinic, any MD methods developed to model it can also be applied to study interfaces in other crystalline materials, such as other common organic HEs, pharmaceuticals, and organic semiconductors. Second, TATB’s highly anisotropic crystal structure is expected to exaggerate anisotropy of its interfacial properties.

**Figure 1:**
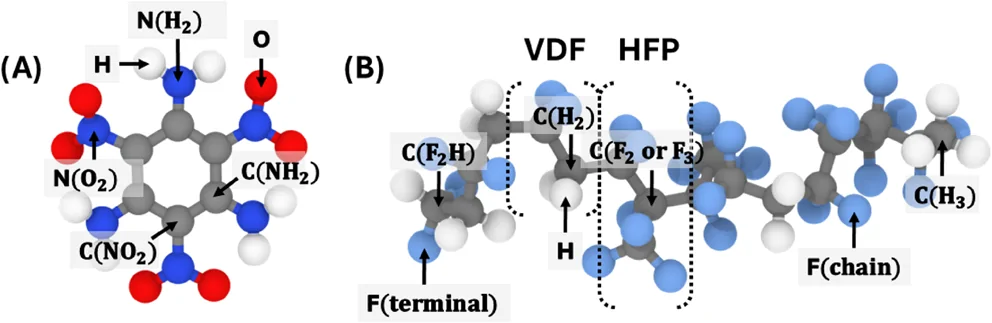
Ball-and-stick representations of (A) a TATB molecule and (B) an oligomer of Ultraflex, a VDF:HFP random copolymer. Atom element types are colored white, gray, dark blue, red, and light blue for H, C, N, O, and F atoms, respectively. Chemically distinct atom types as distinguished by the MD FF are given as annotations.

In what follows, we establish a general work flow that can be applied to predict adhesion properties of molecular crystals and the fundamental cohesive properties of crystal-polymer composites. In Section 2, we establish a classical force field (FF) model for TATB-Ultraflex composites, validate the FF against density functional theory (DFT) calculations, and outline construction protocols to build MD simulations of crystal–crystal and crystal-polymer interfaces. In Section 3, we present a formal analysis of the surface energy expression (eq 1), discuss the regression approach to calculate surface energy, and extend this to calculate interface energy. Practical considerations for evaluating interface energy and its uncertainty in systems with kinetically hindered dynamics typical of polymers are also discussed. Finally, in Section 4, we quantify the energetics of microstructural surfaces and interfaces in TATB-Ultraflex composites and connect these properties to the structure and chemical interactions at the crystal-polymer interface. Our calculations reveal that TATB-Ultraflex PBXs are subject to a range of complex energetic drivers, including for grain coarsening and a greater affinity for TATB crystallites to adhere to each other than to the polymer binder.

## 2 Methods

### 2.1 DFT Calculations

DFT calculations were performed using the CP2K code^41^ to determine mixing rules for describing nonbonded interactions between TATB and Ultraflex in a classical MD FF model. All DFT calculations were performed with the Perdew–Burke–Ernzerhof (PBE) functional^42^ and include D3 dispersion corrections.^43^ The electronic structure was evaluated with Geodecker-Teter-Hutter pseudopotentials^44^ and the DZVP-MOLOPT-GTH basis set^45^ using Γ-point sampling without spin polarization. A 400 Ry cutoff was used for the finest grid and the self-consistent field convergence threshold was set to 1 × 10^–6^ H. All calculations involved pseudoisolated TATB-Ultraflex dimer configurations and used a large 35 × 20 × 20 Å^3^ 3D periodic cell with excess vacuum to reduce self-interactions through the periodic boundary.

### 2.2 Classical MD Simulation Details

All-atom classical MD simulations were performed using the massively parallel software LAMMPS^46^ with GPU acceleration via KOKKOS.^47^ Trajectory snapshots were rendered using the OVITO^48^ software package. TATB was modeled using the recently developed nonreactive CEA-LLNL-Missouri force field (CLM FF),^49^ which has a class-I functional form and has been extensively validated against TATB crystal thermomechanical and thermodynamic properties. Ultraflex was modeled using the Polymer Consistent Force Field, Enhanced version (PCFF+),^50^ which uses a class-II functional form. The PCFF+ model has been shown to accurately reproduce polymer densities for a variety of polymers such as poly­(methyl methacralate) and polyisobutylene.^51^ Cross interactions between TATB and Ultraflex were modeled using standard interaction potentials and mixing rules that are described below. Nonbonded repulsion and dispersion terms were evaluated in real space with an 11 Å cutoff and Coulomb electrostatic interactions were evaluated between fixed atom-centered partial charges with the Wolf potential^52^ using an 11 Å cutoff. Simulation cell constructions involving TATB and Ultraflex are described in detail in subsequent sections.

Trajectories were integrated using Martyna-Tobias-Klein equations of motion (EOM),^53^ which include options for a Nosé-Hoover style thermostat and barostat. Time stepping was performed using the velocity-Verlet integrator^54^ with a time step of 1 fs, but we reduced this to 0.5 fs in some system preparation steps when configurations were far from equilibrium. Simulations to prepare the free surface and interface configurations used either isochoric-isothermal (*NVT*) or isobaric–isothermal (*NPT*) EOM with the thermostat and barostat damping parameters respectively set to 100 and 1000 fs. Production calculations of surface and interface energies were performed with *NVT* dynamics using a Langevin thermostat^55,56^ with a damping time of 100 fs to attenuate long-lived correlations (see SI for further discussion). Unless otherwise noted, simulations of pure polymer used an isotropic barostat and simulations involving crystal were performed using a triclinic barostat.

Nonbonded repulsion and dispersion interactions between TATB and Ultraflex were modeled with standard Lorentz–Berthelot (L-B) mixing rules^57,58^ to mix the pairwise Lennard-Jones (L-J) terms. The CLM FF utilizes a 12–6 L-J form with Coulomb electrostatics

2 $$E=4\epsilon_{\alpha \beta }\left[{\left(\frac{\sigma_{\alpha \beta }}{r}\right)}^{12}-{\left(\frac{\sigma_{\alpha \beta }}{r}\right)}^{6}\right]+\frac{q_{\alpha }q_{\beta }}{4\pi \epsilon_{0}r}$$

and PCFF+ utilizes a 9–6 L-J form with Coulomb term for the nonbonded interactions

3 $$E=\epsilon_{\alpha \beta }\left[2{\left(\frac{\sigma_{\alpha \beta }}{r}\right)}^{9}-3{\left(\frac{\sigma_{\alpha \beta }}{r}\right)}^{6}\right]+\frac{q_{\alpha }q_{\beta }}{4\pi \epsilon_{0}r}$$

Here, *r* is the pairwise distance between two atoms of types α and β, parameters ϵ and σ control the well depth and position, the *q* are atom-centered constant partial charges, and ϵ_0_ is the permittivity of vacuum. Homoatom values for ϵ and σ are collected in Table 1 with FF atom types defined in Figure 1. Heteroatom TATB–TATB interactions with the CLM FF are computed using geometric means and heteroatom Ultraflex-Ultraflex interactions with PCFF+ are computed using sixth-power mixing rules. We considered these two mixing rule choices in addition to standard L-B mixing rules for TATB-Ultraflex interactions.

**Table 1:** Nonbonded FF Parameters for TATB and Ultraflex

Atom Type	ϵ (kJ/mol)	σ (Å)	*q* (e)
Ultraflex			
C(F_2_ or F_3_)	0.1673	3.7500	0.320
C(F_2_H)	0.1464	3.7500	0.267
C(H_2_)	0.2653	3.7584	–0.106
C(H_3_)	0.2971	3.8840	–0.159
F(terminal)	0.2502	3.2000	–0.160
F(chain)	0.2042	3.4210	–0.160
H	0.0962	2.8780	0.053
TATB			
C(NO_2_)	0.0418	3.8309	0.254
C(NH_2_)	0.0418	3.8309	–0.218
N(O_2_)	0.0418	3.8309	0.690
N(H_2_)	0.0418	3.8309	–0.732
O	2.5104	2.6727	–0.430
H	0.8368	1.5145	0.433

Heteroatom mixing rules for TATB-Ultraflex interactions were chosen based on PBE-D3 DFT calculations of the potential energy surface for dimer interactions between a TATB molecule and an Ultraflex oligomer with eight repeat units (i.e., an 8-mer). Potential energy surfaces were computed through rigid body displacements as a function of *r*_CM_, the displacement between molecular centers of mass. Three dimer orientations were chosen to selectively probe close contacts between Ultraflex and the amine, nitro, and ring groups in TATB. Results from these calculations are shown in Figure 2.

**Figure 2:**
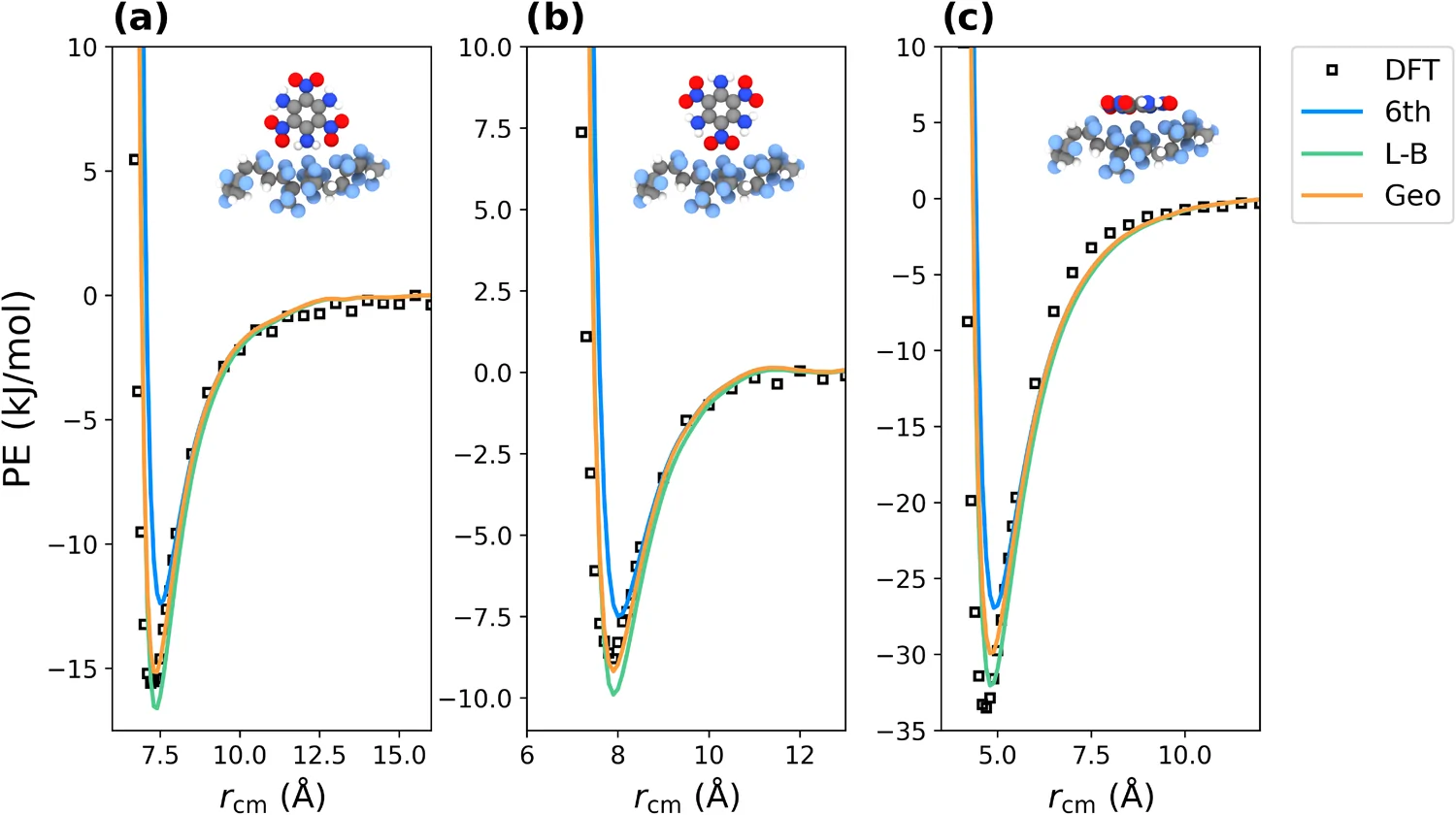
Potential energy surfaces for interactions between a TATB molecule and Ultraflex 8-mer oriented to probe close contacts with the TATB (a) amine group, (b) nitro group, and (c) benzene ring. Dimer orientations are shown as inset snapshots with atoms colored white, gray, dark blue, red, and light blue for H, C, N, O, and F atoms, respectively. Reference PBE-D3 DFT calculations (symbols) are compared with three FF mixing rule choices (curves) that include sixth-power (6th), Lorentz–Berthelot (L-B), and geometric (Geo) means.

As can be seen in the figure, all three potential energy surfaces exhibit attractive wells, with DFT-predicted binding energies that vary between −33.5 kJ·mol^–1^ to −8.8 kJ·mol^–1^. Based only on these calculations, it is reasonable to anticipate that adhesion between TATB and Ultraflex will be energetically favorable (*k*_B_ *T* = 2.5 kJ·mol^–1^ at room temperature). All three mixing rule choices capture the dimer binding energies to within 7 kJ·mol^–1^, but L-B rules are consistently closest to the DFT predictions and differ by no more than 1.5 kJ·mol^–1^. The sixth-power mixing rules consistently underpredict the dimer binding energy whereas the other two mixing rule choices do not exhibit a systematic difference. Accuracy in mixing the CLM FF with PCFF+ is generally better than was obtained with optimized FF mixing rules in previous work that studied TATB-water interactions^59^ and errors are of similar magnitude to the expected accuracy of the PBE-D3 DFT calculations.^60^ For these reasons, we simply adopt L-B mixing rules rather than attempt further optimization of the pairwise interactions.

### 2.3 Bulk Polymer Configurations

Ultraflex polymer was modeled as a 70:30 VDF:HFP random linear copolymer with a number-averaged molecular weight of 12.54 kDa (see Figure 1). An ensemble of initial chain configurations was generated by randomizing the order of the VDF and HFP monomers and FF topology files were prepared using the MedeA 3.7 software package.^61^ In preliminary work,^62^ we considered monodisperse amorphous Ultraflex configurations with polymerization degrees of *N* = 8, 16, 32, 64, and 128. We focus here on monodisperse 128-mer systems. Inspections of the chain dynamics indicate that the 128-mer systems exhibited *N* ^2.6^ scaling, suggesting that the chains are entangled but not fully in the reptation regime^63^ (see SI for further details). The predicted bulk density at 300 K was found to be 1.68 g/cm^3^ by quenching the pure polymer melt to 300 K and 1 atm under *NPT* dynamics at a rate of 20 K/ns. Our bulk density underpredicts the manufacturer’s reported density of 1.815 g/cm^3^.^64^ We also note that the glass transition temperature obtained for Ultraflex using PCFF+ is higher than the value reported in the manufacturer’s datasheet; this discrepancy likely arises from uncertainty in the crystallinity of real samples.^62^ Prior work suggests that commercially available Ultraflex is not an ideal random copolymer, but instead contains poly­(VDF) sequences that form crystalline subdomains.^65^ Although we do not examine crystallinity effects here, similar morphological complexity occurs in other copolymers used as PBX binders,^66^ motivating the development of atomistic modeling methods applicable to such heterogeneous configurations.

Bulk amorphous configurations were prepared through a multistep procedure. First, 100 128-mer Ultraflex chains were placed on lattice sites in a large cubic simulation cell with side length 300 Å. The chains were partially relaxed at 600 K for 500 ps with *NVT* dynamics. Following this initial relaxation, the cell was dynamically and isotropically compressed to a density of 1 g·cm^–3^ over 500 ps with a thermostat set to 600 K. A long 50 ns *NPT* trajectory was then integrated at 600 K and 1 atm, from which we computed the autocorrelation function *C*_EE_(τ) of the chain end-to-end separation vectors **R**_EE_ as

4 $$C_{\mathrm{EE}}\left(\tau \right)=\frac{\left\langle R_{\mathrm{EE}}\left(\tau \right)\cdot R_{\mathrm{EE}}\left(0\right)\right\rangle }{\left\langle R_{\mathrm{EE}}^{2}\left(0\right)\right\rangle }\approx \exp\left[{\left(\frac{\tau }{\tau_{\mathrm{EE}}}\right)}^{\beta }\right]$$

Here, τ is the lag time,⟨⟩ denotes an average over chains and time origins, τ_EE_ is the characteristic time constant, and β is a stretching exponent. We obtained τ_EE_ = 15 ns for Ultraflex 128 mers at 600 K and 1 atm, which we take as a lower bound for the minimum required period when sampling pseudoindependent polymer configurations used for constructing crystal-polymer interfaces.

Melt configurations used for subsequent constructions were prepared at 600 K starting from the initial equilibrated 100-chain configuration. Independent 100-chain configurations were prepared through long *NVT* simulations with the cell dimensions set based on the *NPT*-predicted density ρ = 1.32 g·cm^–3^ at 600 K and 1 atm. Larger melt cells containing 200 and 400 chains were prepared by periodically replicating the base cell along *z*. A small subset of simulation constructions used a smaller 50-chain amorphous configuration. These 50-chain configurations were prepared following the same general procedure as described above, with the only exception being that we compressed the starting cell to the previously determined equilibrium density (skipping the *NPT* phase).

### 2.4 Surface and Interface Configurations

Surface and interface energies were computed using 3D periodic simulation cells that inscribed 2D periodically infinite crystal surfaces, crystal–crystal interfaces, and crystal-polymer interfaces. Snapshots of representative interface systems are given in Figure 3 and construction methodologies are outlined below. All constructions were prepared using the GCCM^36^ with the triclinic *P*1̅ TATB crystal structure determined by Cady and Larson^37^ and the equilibrium lattice parameters for TATB at 300 K and 1 atm predicted by the CLM FF.^49^ The GCCM code works by identifying translational-symmetry preserving crystal cuts and rotations to orient a user-specified direction **S** in the crystal frame with the *z* direction of a simulation cell in a Cartesian lab frame. We consider only the (001) and (011) TATB crystal facets, which are the two lowest-energy facets (as predicted by the CLM FF) that coincide with known TATB crystallographic slip systems.^67^ The (001) and (011) slip planes are structurally dissimilar, whereas the (011) plane is similar to the other three transverse slip planes (011̅), (101), and (1̅01) that break the hydrogen-bonded crystal layers. We chose the (001) and (011) facets as a representative subset for examining the surface and interfacial properties of TATB as a fully general methodological demonstration, rather than as an exhaustive study of the energies associated with all possible crystal facets or roughness on larger length scales. In what follows, we first briefly outline the GCCM approach to build oriented crystals and crystal–crystal interfaces and then describe extensions for constructing crystal-polymer interfaces. Discussion on GCCM algorithms can be found in refs 36 and 38 with additional practical considerations for building crystal–crystal interfaces discussed in ref 69.

**Figure 3:**
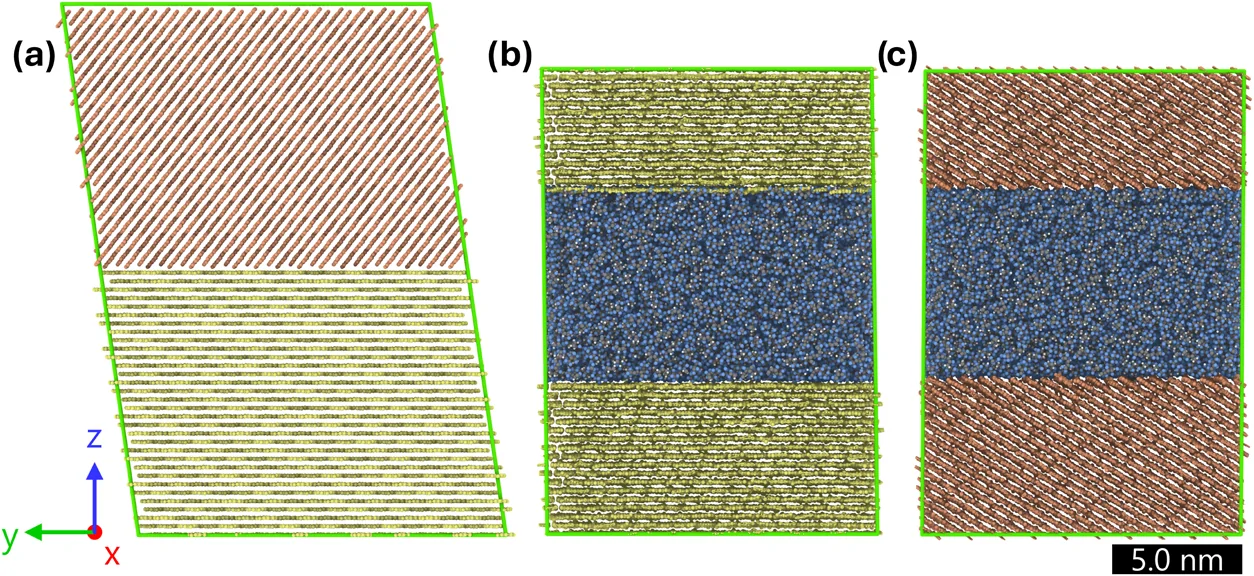
Representative TATB interface constructions, including (a) a crystal–crystal interface between the (001) and (011) facets, (b) a crystal-polymer interface between the (001) facet and Ultraflex, and (c) a crystal-polymer interface between the (011) facet and Ultraflex. TATB atoms in the (001) and (011) oriented crystal grains are respectively colored yellow and orange. Ultraflex atoms are colored white, gray, and light blue, for H, C, and F.

For a generic Bravais lattice with primitive lattice vectors **a**, **b**, and **c**, one can define any arbitrary new symmetry-preserving cell by three edge vectors (**x**_1_, **x**_2_, **x**_3_) provided that those edge vectors are also lattice vectors. That is, we require

5 $$x_{j}=m_{j}a+n_{j}b+p_{j}c$$

where *m*_*j*_, *n*_*j*_ and *p*_*j*_ are integers. The GCCM employs a series of searches over the *m*_1_,...,*p*_3_ integer space to identify cells that satisfy a fundamental alignment constraint

6 $$x_{1}\times x_{2}∥S$$

within a finite tolerance, along with secondary constraints that aim to reduce overall simulation cell size and deviations of the cell angles from 90°. In our constructions, we also specified a second alignment constraint to force **x**_1_ = **a** in all cases. This second alignment constraint imposes an interface twist angle of 0.0° for crystal–crystal interfaces.^68^ By convention, we adopt a Cartesian lab frame for the simulation cell in which we orient **x**_1_ along *x*, **x**_2_ in the *x*-*y* plane, and **x**_3_ in the + *z* half space. This leads to a 2D periodically infinite crystal slab in the *x* and *y* dimensions with the chosen surface/interface normal vector **S** oriented along *z*.

Crystal–crystal interface constructions used the GCCM to identify two commensurate crystal grains defined by (**x**_1_^*A*^, **x**_2_^*A*^, **x**_3_^*A*^) and (**x**_1_^*B*^, **x**_2_^*B*^, **x**_3_^*B*^) with chosen orientation vectors **S** ^*A*^ and **S** ^*B*^. Interfaces were formed by stacking the two crystal grains along **x**_3_ and inscribing both grains in a single simulation cell, which produces two periodically equivalent interfaces. Additional GCCM constraints minimize lattice mismatches in the transverse dimensions (i.e., **x**_1_^*A*^ = **x**_1_^*B*^ and **x**_2_^*A*^ = **x**_2_^*B*^) and the longitudinal direction (i.e., **x̂**_3_^*A*^ = **x̂**_3_^*B*^, where **x̂** denotes a unit vector). In practice, some lattice mismatch is unavoidable and the preceding equalities are held only to within a user-specified strain tolerance. Both crystals were inscribed in a single simulation cell (**x**_1_, **x**_2_, **x**_3_) that averaged the strains imposed on the two crystals, for instance via **x**_1_ = (**x**_1_^*A*^ + **x**_1_^*B*^)/2. Only the orientations of **x̂**_3_^*A*^ and **x̂**_3_^*B*^ are constrained (not lengths), so crystal grains of arbitrary thickness can be formed by replicating the GCCM solution cell for a given crystal along **x**_3_ and scaling the edge vector length *x*_3_ appropriately. Our constructions of TATB(001)-TATB(011) interfaces had no more than 0.33% strain imposed on cell lengths and a 0.3° strain on cell angles; stress along the normal to the crystal–crystal interface plane was relaxed through a barostat coupled to the **x**_3_ dimension.

Crystal-polymer interface constructions involve merging two key components, including (1) a 2D periodically infinite oriented crystal film in a 3D cell and (2) a 2D periodically infinite polymer film in a 3D cell. Our five-step procedure for combining these two films to form an interface is given in Figure 4. This procedure was designed to mimic PBX pressing operations and involves a high-temperature relaxation stage to accelerate polymer chain dynamics followed by a high-temperature, high-pressure compression stage and quenching to ambient conditions. The main stages of this procedure include:

1. *Build Crystal and Polymer*: An oriented and nearly orthorhombic crystal cell was prepared using the GCCM and a 3D periodic polymer melt configuration was obtained following the procedures in Section 2.3. The transverse dimensions of the polymer cell were matched to the transverse dimensions of the crystal cell and vacuum space was introduced to both the crystal and polymer cells along *z* to generate thin films.
2. *Merge and Wet*: While holding the crystal rigid, a small net velocity *v*_*z*_ = −5 m·s^–1^ was added to the polymer film to wet it to the crystal surface through a short *NVT* simulation at 600 K. After successful wetting, the polymer was relaxed at 600 K for 2 ns with *NVT* dynamics while holding the crystal rigid.
3. *Initial Press*: Both the crystal and polymer were thermalized at 500 K (15% below the predicted crystal melting point^49^). The length of **x**_3_ was dynamically contracted over 2 ns with a 500 K thermostat to yield a total volume *V*_tot_ = *V*_TATB_^500 K^ + *V*_Poly_^500 K^. Here, *V*_TATB_^500 K^ and *V*_Poly_^500 K^ are the predicted equilibrium volumes for the crystal and polymer in isolation at 500 K and 1 atm. This results in the formation of two interfaces given the periodic boundary conditions.
4. *Press and Cool*: A 1000 atm barostat was applied along the **x**_3_ dimension only and a 2 ns long trajectory was integrated with a thermostat that was linearly ramped from 500 to 300 K. Application of a high-pressure barostat (as opposed to a 1 atm barostat) was needed to maintain cohesion of the interface during cooling.
5. *Decompress*: While holding the temperature at 300 K, the barostat was linearly ramped down to 1 atm over another 2 ns. Following decompression, long O­(10 ns) *NVT* trajectories were integrated at 300 K with a Langevin thermostat.

**Figure 4:**
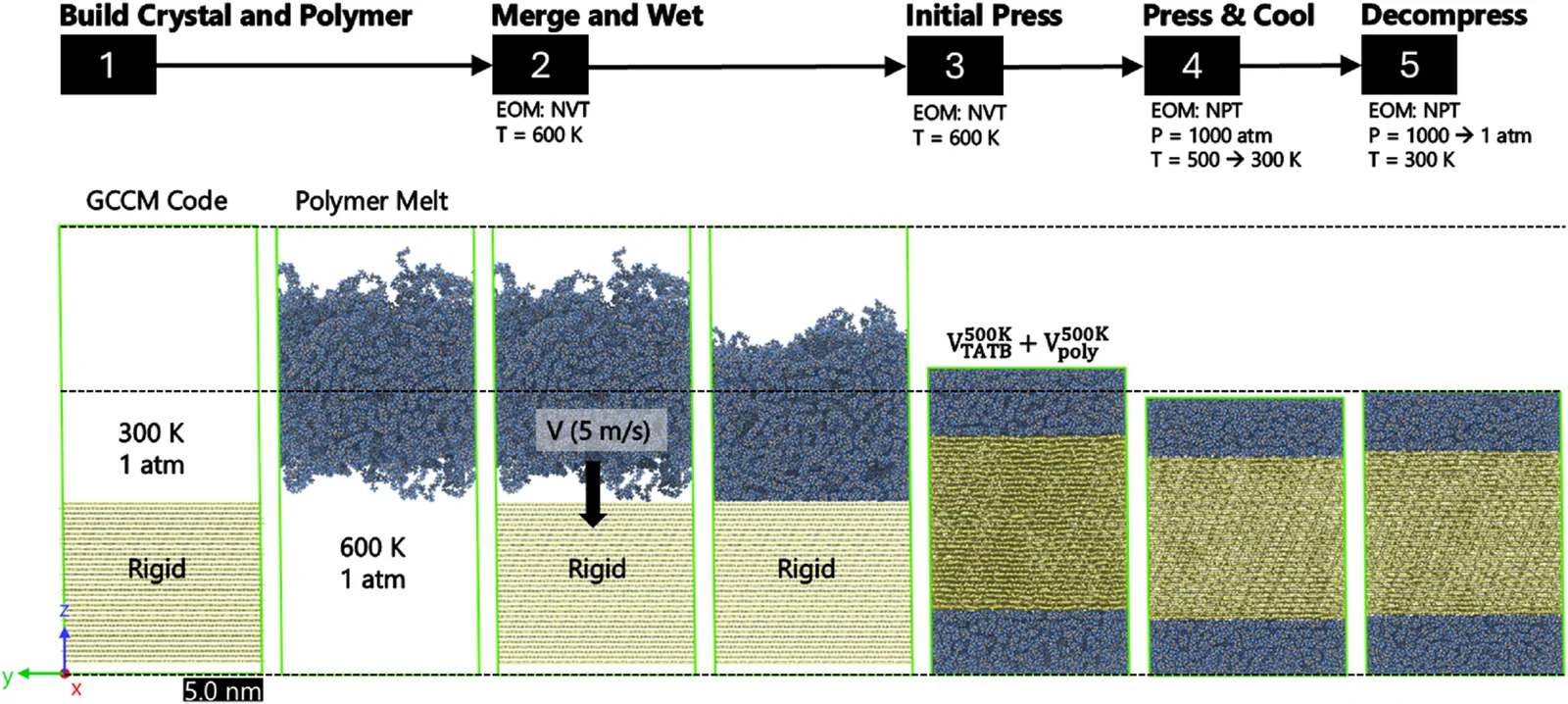
Construction sequence to prepare crystal-polymer interfaces. TATB atoms are colored yellow, Ultraflex atoms are colored white, gray, and light blue, for H, C, and F, and the simulation cell is rendered with green lines. Dynamical EOM and thermostat/barostat targets are specified below each stage header.

## 3 Surface and Interface Energy Calculation Details

### 3.1 Numerical Sensitivity

As discussed in the introduction, it is well-known that surface energy calculations exhibit poor convergence properties,^30^ which can be overcome through a linear regression approach.^31–34^ Here we formally summarize this convergence issue through an analysis of the condition number for the surface energy equation. We then examine and extend the established regression-based approach for surface energy calculations to arrive at an analogous expression for the interface energy.

The surface energy γ is defined as the amount of energy per unit area required to expose bulk to a vacuum, which can be calculated through MD-derived energy values using eq 1 in Section 1. Thus, defined, one can calculate γ through two separate MD simulations that respectively sample bulk and surface configurations to obtain *E*_bulk_ and *E*_surf_. We refer to this method of calculating γ as the “direct approach.” At least two issues arise in practice when evaluating γ through the direct approach. The first and most-significant issue is that the magnitudes of *E*_bulk_ and *E*_surf_ are very similar and the difference between these two quantities is typically many orders of magnitude smaller. This leads to poor numerical conditioning, meaning small changes (or errors) in the input result in large relative changes in the output. The second issue is that *E*_surf_ is highly correlated with *E*_bulk_ (i.e., *E*_surf_ closely tracks *E*_bulk_ as they both scale linearly with *N*_mol_). These correlations must be accounted for when assessing uncertainties.

Poor numerical conditioning can be conveniently summarized via a condition number κ, which is defined as the relative change of the output (γ) given some perturbation of the inputs (*E*_surf_ and *E*_bulk_). Here, perturbations in *E*_surf_ and *E*_bulk_ represent uncertainty and/or error in the computed input energies from separate simulations, not physical variation in the bulk state. We compute the condition number utilizing an *L*_2_ norm for the perturbations in *E*_surf_ and *E*_bulk_ as

7 $$\kappa \left(E_{\mathrm{surf}},E_{\mathrm{bulk}}\right)=\frac{\sqrt{{\left(E_{\mathrm{surf}}\frac{\partial \gamma }{\partial E_{\mathrm{surf}}}\right)}^{2}+{\left(E_{\mathrm{bulk}}\frac{\partial \gamma }{\partial E_{\mathrm{bulk}}}\right)}^{2}}}{\left|\gamma \left(E_{\mathrm{surf}},E_{\mathrm{bulk}}\right)\right|}$$

which reduces to

8 $$\kappa \left(E_{\mathrm{surf}},E_{\mathrm{bulk}}\right)=\frac{\sqrt{E_{\mathrm{bulk}}^{2}+E_{\mathrm{surf}}^{2}}}{\left|E_{\mathrm{surf}}-E_{\mathrm{bulk}}\right|}$$

The condition number reflects a worst-case scenario in the propagation of uncertainties in the input parameters. Small condition numbers (κ ≤ 1) indicate low sensitivity to input uncertainties.

As a concrete example, we applied the above condition number analysis to assess sensitivity of the predicted TATB (001) facet surface energy as a function of system size. We created 8 TATB free surface configurations with equivalent transverse dimensions of 12.5 × 12.4 nm^2^ and different numbers of TATB crystal layers along *z*, which ranged from 3.3 nm (1080 molecules) up to 40 nm (28672 molecules). The minimum crystal slab thickness was selected to be three times larger than the FF cutoff distance to prevent direct interactions between the two surfaces and surface reconstruction regions. The energy of each surface configuration *E*_surf_ was obtained via a conjugate gradient energy minimization. The bulk energy per molecule *E*_bulk_^mol^ was obtained from a 20 nm sized system without free surfaces via minimization, and this was used to obtain the bulk reference energy for each of the four different sized systems as *E*_bulk_ = *N*_mol_ *E*_bulk_^mol^.

Figure 5(a) shows the condition number κ plotted as a function of the ratio *R* = *E*_surf_/*E*_bulk_, with specific values shown for the 5, 10, 20, and 40 nm thick TATB films. Note that *R* → 1 as the system thickness increases. As the system size increases, the absolute difference between *E*_bulk_ and *E*_surf_ remains constant, but the *relative* difference (as compared to *E*_bulk_) approaches zero. Consequently, the condition number asymptotically diverges with increasing size and is orders of magnitude larger than unity for even modest-sized crystal cells. Given a small error δ in *E*_bulk_^mol^, it can be clearly seen from

9 $$\gamma =\frac{E_{\mathrm{surf}}\left(N_{\mathrm{mol}}\right)-N_{\mathrm{mol}}\left(E_{\mathrm{bulk}}^{\mathrm{mol}}+\delta \right)}{2A}$$

that the error magnifies linearly with system size *N*_mol_. This error may arise from many sources of uncertainty, such as numerical noise, systematic error in missing a global minimum, or standard error in a thermal average energy. It is also understood that *E*_surf_ carries uncertainty, but its impact on γ is less significant, especially in situations when a small simulation is used to determine *E*_bulk_^mol^. Figure 5(b) shows the resulting spread in predicted γ for selected errors δ. As can be seen in the figure, *E*_bulk_^mol^ must be known to very high precision (at least 1 × 10^–6^ *E*_bulk_^mol^) for system sizes on the order of 5–50 nm. While smaller cells can be employed for crystalline systems, cells of this size range are needed for polymers. For instance, in our simulations the radius of gyration of an Ultraflex 128-mer is 2.4 nm, which sets a lower limit to the minimum polymer film thickness needed to reach a well-defined bulk state.

**Figure 5:**
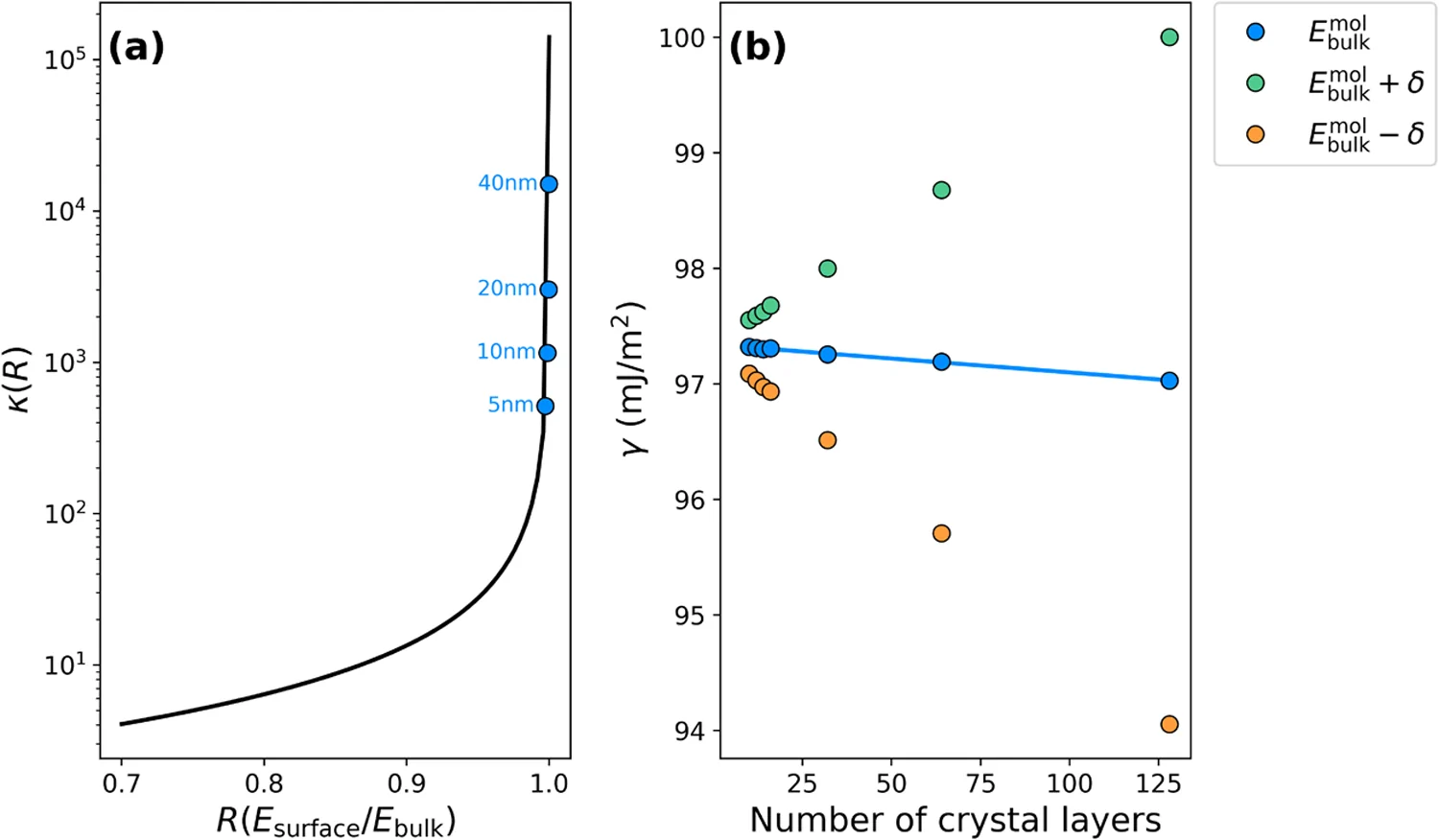
(a) Condition number for the surface energy of a TATB (001) crystal facet plotted as a function of *R* = *E*_surf_/*E*_bulk_. (b) Surface energy of the TATB (001) crystal facet plotted as a function of number of crystal layers, where γ is calculated via the “direct approach” for two cases: (1) with *E*_bulk_ = *N*_mol_ × *E*_bulk_^mol^ and (2) with *E*_bulk_ = *N*_mol_ × *E*_bulk_^mol^ ± δ where δ = 1 × 10^–5^ *E*_bulk_^mol^.

Linear regression offers an alternative, but computationally more intensive, path to compute γ that avoids poor conditioning of the direct approach. Rearranging eq 1 as

10 $$E_{\mathrm{surf}}\left(N_{\mathrm{mol}}\right)=E_{\mathrm{bulk}}^{\mathrm{mol}}\cdot N_{\mathrm{mol}}+2A\gamma$$

yields a linear expression between the energy of a surface system *E*_surf_(*N*_mol_) as a function of the system size *N*_mol_. Thus, framed, γ and *E*_bulk_^mol^ can be obtained as fitting parameters in a linear regression over multiple determinations of *E*_surf_ obtained from MD simulations performed with surface cells of different thin film thicknesses and fixed cross-section *A*. That is, *E*_bulk_^mol^ can be obtained as the slope and 2*A*γ as the intercept term from a (weighted) least-squares linear regression. Both *E*_bulk_^mol^ and γ have uncertainty stemming from uncertainty in *E*_surf_, which is propagated through the linear regression. The main advantage of this approach is that it effectively averages over multiple simulations to mitigate the uncertainty in *E*_bulk_^mol^ that otherwise scales linearly with system size in the direct approach.

For similar reasons as above, numerical conditioning also affects calculations of the interface energy Γ, which is defined as the energy required to form interfaces relative to the two bulk materials as

11 $$\Gamma =\frac{E_{\mathrm{int}}-\left(E_{\mathrm{bulk},1}+E_{\mathrm{bulk},2}\right)}{2A}$$

Here, *E*_bulk,1_ is the potential energy of a 3D-periodic system of material one with *N*_mol,1_ molecules at equilibrium, *E*_bulk,2_ is the analogous energy for material two with *N*_mol,2_ molecules, *E*_int_ is the potential energy of an interface system containing *N*_mol,1_ molecules of material one and *N*_mol,2_ molecules of material two at equilibrium, and *A* is the cross-sectional area of the interface with a factor of 2 arising due to the formation of two interfaces with periodic boundary conditions. In direct analogy with the surface energy calculations, eq 11 can be rearranged as

12 $$E_{\mathrm{int}}\left(E_{\mathrm{bulk},1}^{\mathrm{mol}},E_{\mathrm{bulk},2}^{\mathrm{mol}}\right)=E_{\mathrm{bulk},1}^{\mathrm{mol}}\cdot N_{\mathrm{mol},1}+E_{\mathrm{bulk},2}^{\mathrm{mol}}\cdot N_{\mathrm{mol},2}+2A\Gamma$$

which enables *E*_bulk,1_^mol^, *E*_bulk,2_^mol^, and Γ to be determined from a multilinear regression. As with the surface energy calculation, the regression approach averages over results from multiple MD simulations of interface systems with different thin film thicknesses and fixed cross-section *A*. However, both *N*_mol,1_ and *N*_mol,2_ must be independently varied to compute the variance-covariance matrix in the regression, which requires the construction of independent interface systems of varying sizes.

### 3.2 Data Sampling

Surface (γ) and interface (Γ) energies were computed at finite temperature using the regression approach described just above with average potential energy values and corresponding uncertainties that were determined from long MD simulations. Because we use a classical MD FF, it is straightforward to split the total potential energy into separate components

13 $$E_{\mathrm{Tot}}=E_{\mathrm{VDW}}+E_{\mathrm{Mol}}+E_{\mathrm{Coul}}$$

where *E*_Mol_ is the contribution from intramolecular bonded interactions (bonds, angles, and dihedrals), *E*_VDW_ is the contribution from van der Waals interactions (all L-J potential terms), and *E*_Coul_ is the contribution from Coulomb electrostatics. This partitioning allows for a detailed inspection of the atomic interactions that contribute to surface and interface energies. Given the high degree of numerical sensitivity in surface/interface energy calculations (even with regression), we discuss here our determinations of input energy values for these calculations and their impact on γ and Γ.

All MD simulations used to sample energies were performed using *NVT* dynamics with a Langevin thermostat in which the energy time series was initially sampled every 100 fs. Using a Langevin thermostat with an appropriate τ_damp_ allows for a shorter correlation time in the energy time series, allowing for more statistically independent samples to be extracted from a given trajectory length. We determined the correlation time in the energy time series to be <2 ps in our systems (see SI for further discussion). Based on this correlation time, we downsampled the time series and took (pseudo)­independent measurements every 2 ps. Averages ⟨*x*⟩ were obtained by blocking the downsampled time series and the standard error of the mean was taken as the uncertainty (i.e., $\sigma_{\left\langle x\right\rangle }=\sigma_{x}/\sqrt{n}$ where σ_*x*_ is the sample standard deviation of sample size *n*). An analogous *NPT*-like protocol was also considered in which we imposed a barostat along *z*. As will be discussed below and in the SI, Γ is insensitive to the choice of *NVT* or *NPT* dynamics.

Figure 6 shows *E*_Tot_, the pressure, and σ_*zz*_ (the stress normal to the interface) for three representative interface systems over 10 ns. There is a clear decrease in *E*_Tot_ for the TATB-Ultraflex interfaces whereas the TATB(001)-TATB(011) crystal–crystal interface remains steady over time. The TATB-Ultraflex interfaces also start in a state of tension, which quickly relaxes (if incompletely) before beginning to plateau after 5 ns. This tensile state is driven by stresses normal to the interface clearly seen in σ_*zz*_, despite this tensile state there is no evidence of bulk void formation. Similar results were obtained for Ultraflex thin films exposed to vacuum (see SI). In contrast, the crystal–crystal interface system stresses are steady in time, but are nonhydrostatic due to the strains that must be imposed on each crystal grain to inscribe the interface in a single periodic cell. The *NPT* simulation series (not shown) exhibited a similar relaxation of *E*_Tot_ with the **x**_3_ cell length evolving in time.

**Figure 6:**
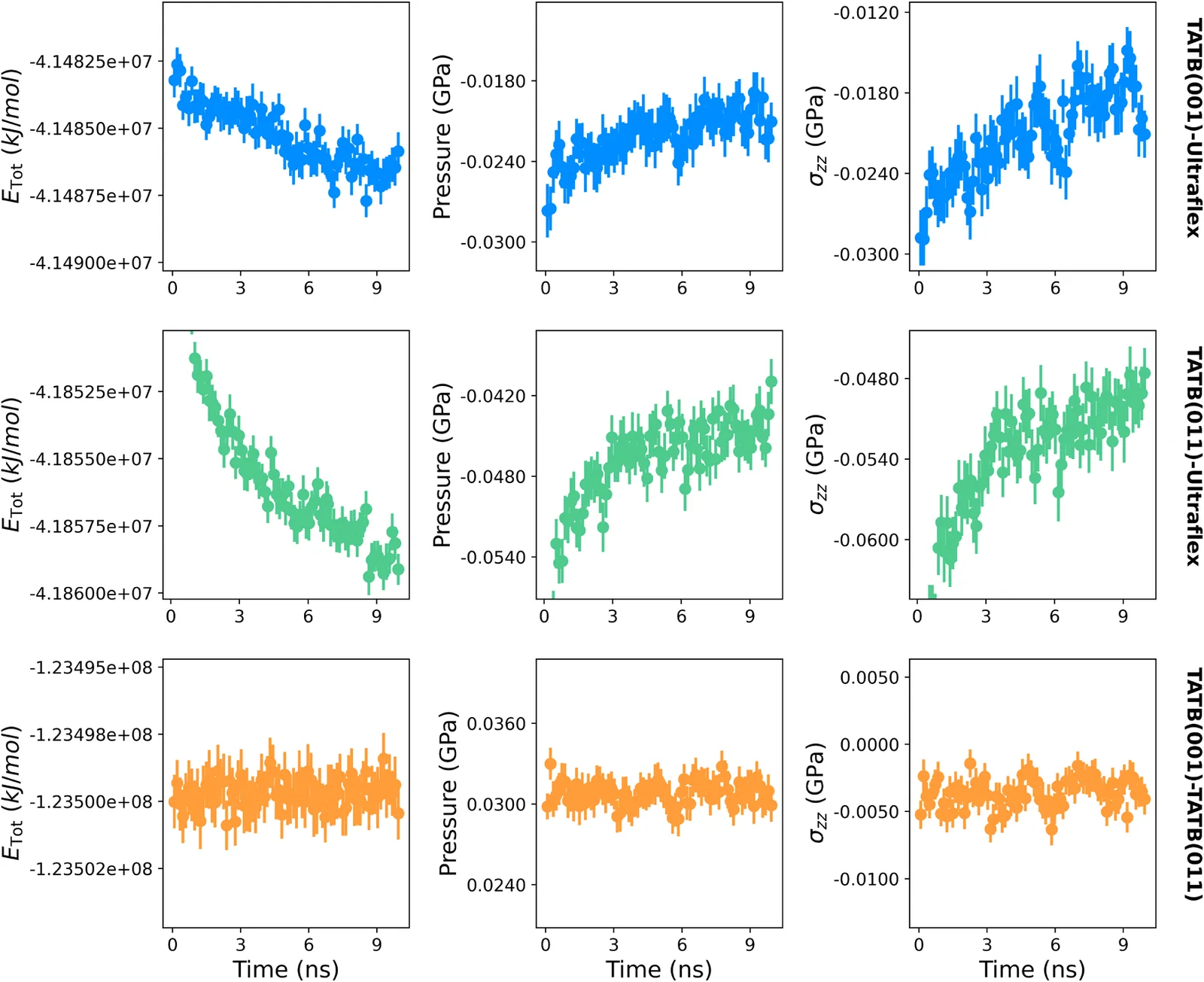
Block-averaged time series of potential energy (first column), pressure (second column), and σ_*zz*_ for three representative interface systems under *NVT* dynamics obtained using a block window of 128 ps. The specific systems include TATB(001)-Ultraflex (top row, blue), TATB(011)-Ultraflex (middle row, green), and TATB(001)-TATB(011) (bottom row, orange).

From these results, it is clear that the interface systems involving Ultraflex exhibit very long time scale relaxation processes that occur on >10 ns time scales with either *NVT* or *NPT*-like dynamics. Long-time scale relaxation dynamics is typical of most polymers and performing very long (>100 ns) MD simulations with an all-atom model is not generally feasible, especially since multiple independent MD simulations are required to compute γ and Γ with the regression approach. At the same time, it is unclear from the above results whether these polymer relaxation processes influence the main quantities of interest (γ and Γ).

To this end, we assess Γ and the bulk energies *E*_bulk_^mol^ derived from the regression approach as functions of time. The input *E*_int_ values were determined within 128 ps long time blocks, which is short relative to the relaxation time scale but large enough to obtain good statistics considering the 2 ps fundamental sampling period. This choice was found to minimize overall propagated uncertainties in Γ, given that as the number of blocks *N*_b_ increases, there are more independent samples of *E*_int_, but these are known with less precision due to having fewer pseudoindependent data points *n* in each block (see SI for further discussion). A regression calculation was performed on each block in the series to obtain Γ­(*t*) and *E*_bulk_^mol^(*t*), which are plotted for the three different interface system types in Figure 7. Analogous plots for Γ derived from *NPT* simulations and for surface energy calculations are given in the SI.

**Figure 7:**
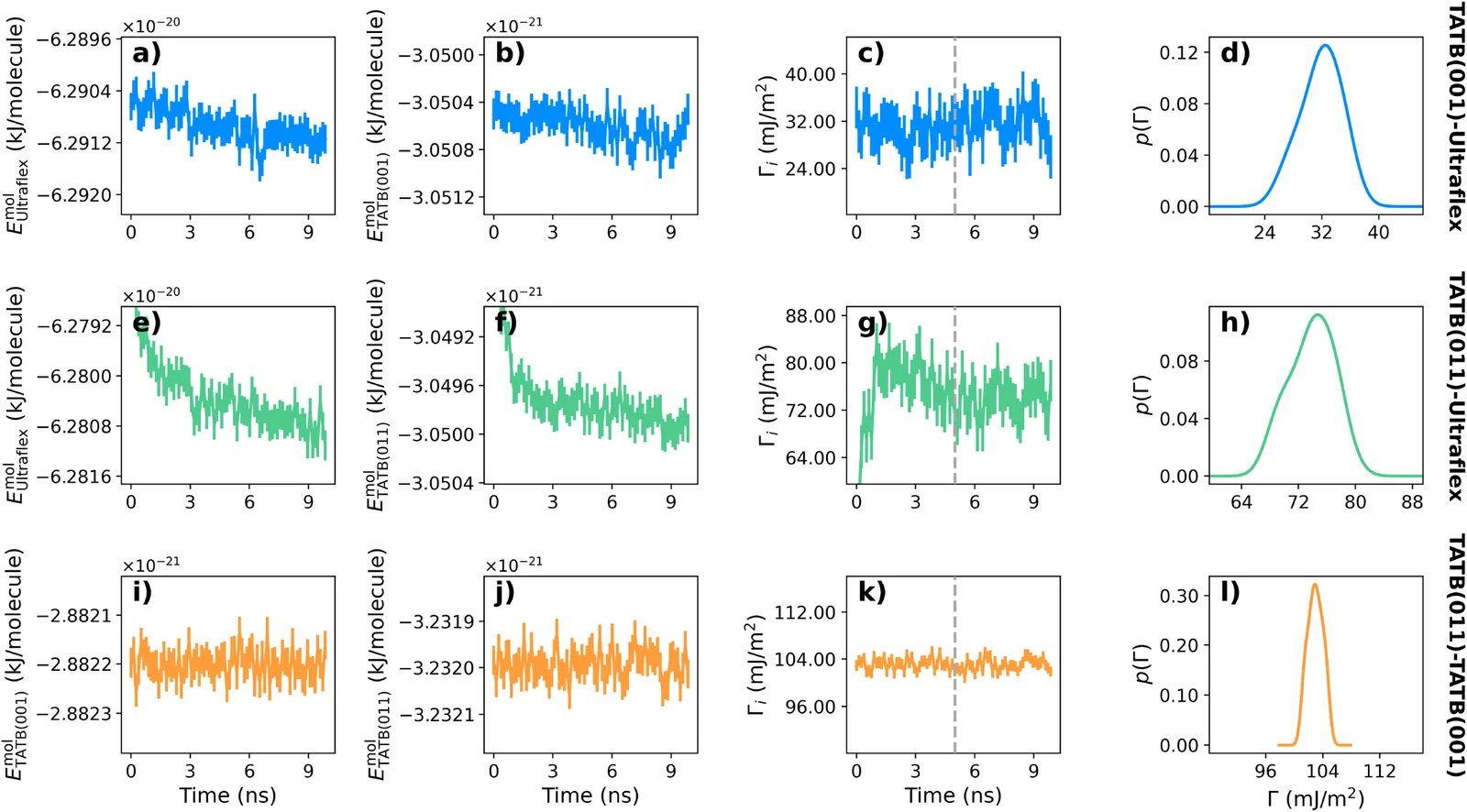
Time histories and sampling distributions for the TATB(001)-Ultraflex interface (top row), the TATB(011)-Ultraflex interface (middle row), and the TATB(001)-TATB(011) interface (bottom row). Panels (a) and (e) show the bulk energy of Ultraflex; panels (b) and (i) show the bulk energy of TATB(001); panels (f) and (j) show the bulk energy of TATB(011); panels (c), (g), and (k) show the time-dependent interface energy Γ_*i*_, with the gray dashed line marking the end of the equilibration period; and panels (d), (h), and (l) show the corresponding sampling distributions *p*(Γ). The sampling distributions were calculated over the final 5 ns of Γ_*i*_ according to eq 14. The *y*-axis limits for *E*_TATB_^mol^ and *E*_Ultraflex_^mol^ are set by the largest standard deviation among the fitted per-molecule energies.

Focusing on the bulk energies in the first two columns, several features are apparent. First, the bulk energies of both the polymer and the crystal in crystal-polymer interface systems exhibit large uncertainty, whereas the uncertainty is comparatively small in the crystal–crystal interface system. Unsteadiness in time is also apparent in the crystal-polymer systems, especially for the TATB(011)-Ultraflex case, which exhibits a decrease (i.e., relaxation) in time. It is perhaps unexpected that both the polymer and crystal bulk energy can evolve in time, but this has a plausible physical origin. Because σ_*zz*_ in the crystal and polymer films remains in equilibrium at a given point in time, a time-dependent σ_*zz*_ implies that the crystal is strained over time as the polymer relaxes. Contrasting the polymer-containing systems, the crystal–crystal interface system values are steady in time.

Unlike the bulk energies, the interface energies Γ­(*t*) in column three are steady in time for the crystal–crystal systems and after some initial transience the crystal-polymer systems are mostly steady in time with fluctuations in the range of ±5 mJ/m^2^. This fact, coupled with the time-dependent bulk energies, indicates that the evolution of the total energy of each interface system seen in Figure 6 is largely driven by configuration relaxations in bulk rather than at the interface. If we consider each regression calculation on block *i* as yielding a value Γ_*i*_ (with propagated uncertainty σ_Γ,*i*_) as sampling from the same distribution, then the sampling distribution for Γ can be calculated as

14 $$p\left(\Gamma \right)=\frac{1}{N_{b}}\sum_{i=1}^{N_{b}}\frac{1}{\sqrt{2\pi \sigma_{\Gamma ,i}^{2}}}\exp\left[-\frac{{\left(\Gamma -\Gamma_{i}\right)}^{2}}{2\sigma_{\Gamma ,i}^{2}}\right]$$

We computed *p*(Γ) over the last 5 ns of the 10 ns trajectories, yielding the distributions shown in the last column of Figure 7. Analogous plots with other choices for blocking window size are given in the SI. In all cases, we report γ and Γ values with a 95% confidence interval taken from the empirically estimated sampling distribution.

From these calculations, it is clear that forming a crystal–crystal interface from the bulk incurs a larger energetic penalty compared to forming crystal-polymer interfaces. Here, the TATB(001)-TATB(011) interface yields Γ = 102.98_–2.07_^+2.07^ mJ/m^2^, the TATB(001)-Ultraflex interface has the smallest Γ = 32.08_–6.66_^+5.51^ mJ/m^2^, and the TATB(011)-Ultraflex interface Γ = 74.07_–6.99_^+5.96^ mJ/m^2^ is intermediate between these values. While the present values were obtained from *NVT* simulations, essentially identical results were obtained with *NPT* simulations, which are discussed in the SI.

Long-time scale relaxations in the polymer are not desirable, but it can be exceedingly difficult to prepare equilibrium polymer configurations, especially for heterogeneous systems such as crystal-polymer interface constructions. The above analysis gives strong numerical evidence that reliable estimates of the surface and interface energies can be obtained from these systems despite this complication. It also has plausible physical origins. Polymer chains at a crystal interface are very likely locked into a particular configuration that cannot relax even on long time scales. At the same time, the relaxation dynamics of polymer chains at a free surface are considerably faster than bulk due to greater available molecular volume. We note that our calculations use an internal-energy-based definition of the surface and interface energies rather than a free-energy definition and therefore do not include the *T*Δ*S* contribution. Although the finite-temperature MD simulations account for configurational sampling when evaluating the ensemble-averaged internal energy, they do not explicitly evaluate the corresponding entropy contribution.

## 4 TATB-Ultraflex Composites

### 4.1 Surface and Interface Energies

Having established that the regression approach can yield steady-fluctuating surface and interface energy values, even when the underlying system dynamics are not at steady state, we now turn to more-detailed physical inspections of these systems. Experimental techniques to determine surface energies such as contact angle measurement, the Wilhelmy plate method, and IGC often partition the surface energies into two distinct terms, namely the polar component and the dispersion component. An analogous partitioning can be performed using classical FFs, since the total energy is obtained as a sum over contributions from separable interaction terms (see eq 13). Using these partitioned energies, we computed sampling distributions for the “total” γ and Γ as well as contributions from L-J van der Waals interactions *E*_VDW_, Coulomb electrostatics *E*_Coul_, and the molecular covalent terms *E*_Mol_. We also compute the adhesion energy which we define as

15 $$\Gamma^{\prime }=\Gamma -\left(\gamma_{\mathrm{surf},1}+\gamma_{\mathrm{surf},2}\right)$$

which measures the energetic favorability of forming an interface starting from systems with pre-exposed surfaces. Real crystals and polymers exhibit surfaces, so Γ′ is a better measure for ranking energetic favorability to form (or destroy) interfaces than Γ.

Figure 8 shows calculated γ (top row), Γ (middle row), and Γ′ (bottom row) energies for pure and composite TATB-Ultraflex systems with individual contributions due to the three interaction types (see SI for tabulated values). Polymer surface energies were obtained from configurations derived from interface constructions. In these simulations, the crystal molecules were deleted to expose the polymer to vacuum and the resulting thin films were equilibrated with *NVT* dynamics. Hence, there are two separate values for the Ultraflex surface energy, with one derived from an interface with the TATB (001) grain and the other with the (011) grain.

**Figure 8:**
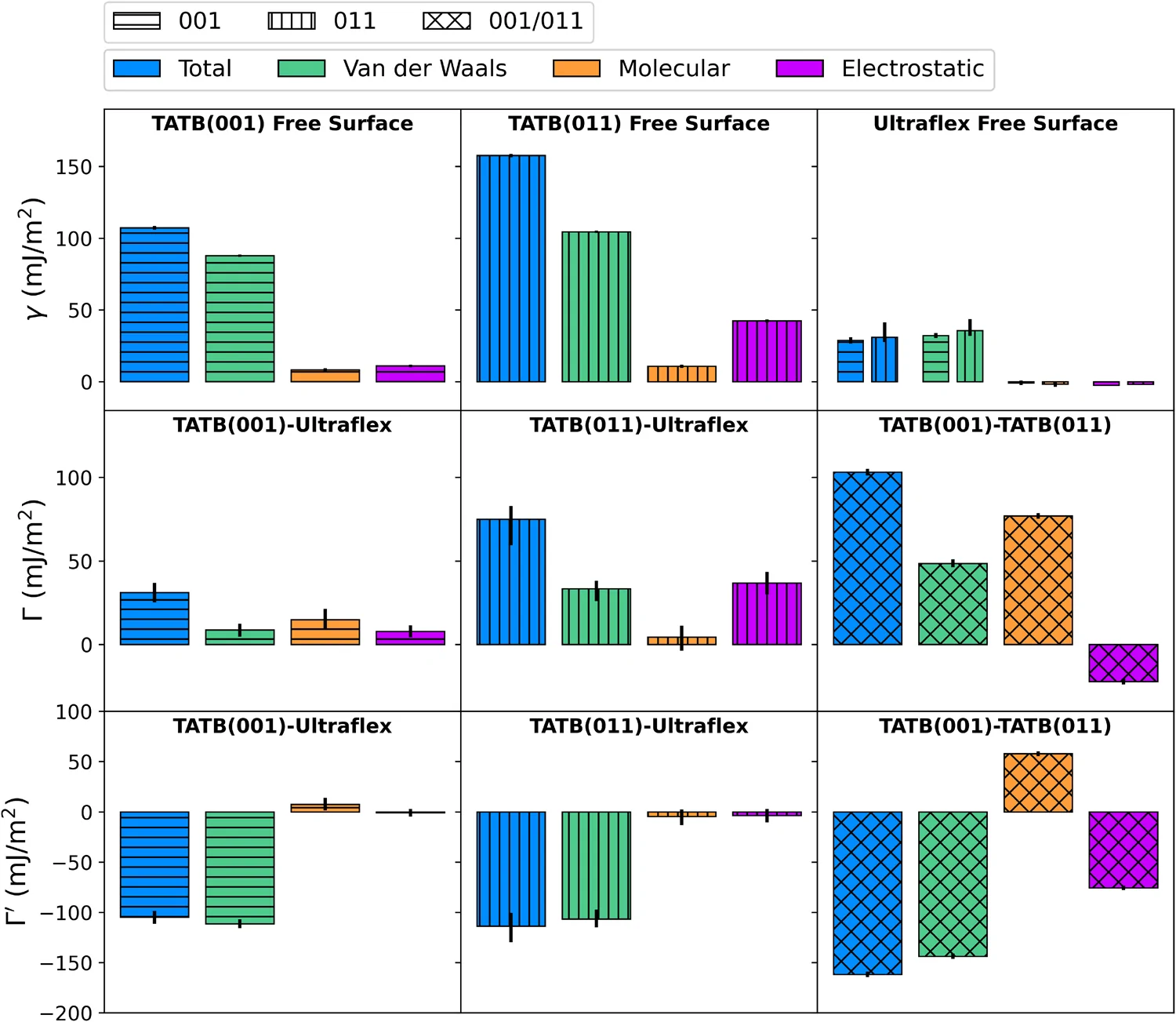
Interaction contributions to the surface energy γ (top row), interface energy Γ (middle row), and adhesion energy Γ′ (bottom row) for TATB-Ultraflex composites. Error bars denote 95% confidence intervals.

All total surface energies are positive. This indicates that both TATB surfaces and the Ultraflex surface are energetically unfavorable relative to the bulk reference state. Similarly, the total interface energies Γ in the second row are also positive, which indicates that forming interfaces is also unfavorable relative to the bulk reference state. Contrasting this are the total adhesion energies Γ′, which are all negative. This indicates that TATB and Ultraflex will favorably adhere if free surfaces are brought into contact. In addition to these high-level assessments, there are numerous features and physical trends of note.

Focusing first on the surface energies γ, it is clear that both TATB facets exhibit a stronger penalty to forming free surfaces compared to Ultraflex (γ_TATB_ ≥ 3.5 γ_UF_). A low surface energy for Ultraflex that is dominated by van der Waals interactions is not unexpected, seeing as many fluoropolymers find application as lubricants and low-stick surface coatings due to these weak interactions.^75,76^ The TATB (011) facet is less energetically stable in comparison to the TATB (001) facet. This is consistent with the plate-like morphology of specially prepared high-quality TATB crystals, which typically express the (001) basal facet.^77–79^ While both crystal surface energies are dominated by van der Waals interactions, the (011) facet exhibits a significant contribution (27%) from Coulombic electrostatics. This is likely because forming an (011) surface breaks the intermolecular hydrogen-bond network that exists within the TATB crystal layers, whereas forming an (001) surface does not break hydrogen bonds.

TATB surface energies have been measured experimentally and predicted through MD in multiple studies. Table 2 collects these literature values from both simulations and experiments, which we discuss in turn.

**Table 2:** Tabulated Literature Values from Experiment and Simulation for Surface Energies of TATB

Surface Type	Method	γ (mJ·m^–2^)	refs
TATB powder	Wilhelmy plate	36.5	^70^
TATB powder	Contact angle	49.49	^71^
TATB powder	Contact angle	51.42	^72^
TATB powder	Contact angle	45.49	^73^
TATB powder	Contact angle	38.7	^74^
TATB powder	Contact angle	51.67	^10^
TATB(001)	MD, Gee FF	66.0	^24^
TATB(001)	MD, ReaxFF-lg	96.7	^33^
TATB(001)	MD, ReaxFF-lg	90.8	^29^
TATB(001)	MD, CLM FF	107.06_–1.12_^+1.16^	This work
TATB(011)	MD, CLM FF	157.65_–1.04_^+1.31^	This work

Several MD calculations of the TATB (001) facet surface energy have been reported that use other FFs and methods. Three values can be directly compared to assess the sensitivity of the calculated values to the choice of MD FF within a consistent choice of calculation approach and crystal facet. Specifically, both Bidault et al.^33^ and Brahmbhatt and Chaudhuri^29^ used the ReaxFF-lg potential^80^ to compute the TATB (001) surface energy using the same regression approach we applied in our own calculations. Both ReaxFF-lg values are similar, if slightly smaller in magnitude (by <17%) to what we obtained with the CLM FF. It is perhaps surprising that the CLM FF and ReaxFF-lg values are so similar, given that ReaxFF-lg substantially over predicts TATB crystal binding energy by 74% relative to experiment and by 104% relative to the CLM FF.^49^ Brahmbhatt and Chaudhuri^29^ also characterized the surface free energy using nonequilibrium thermodynamic integration, explicitly accounting for the *T*Δ*S* contribution, and obtained 64.7 mJ·m^–2^; this indicates that the *T*Δ*S* contribution is 26.1 mJ·m^–2^. One of the main conclusions of their work, which inspected four different molecular crystals, is that the entropy contribution to surface energy is on the order of 30%. While modest, this contribution is not negligible. The TATB surface free energy calculations by Gee et al. in their 2007 report^24^ used the Gee et al. TATB FF^81^ originally published in 2004. The CLM FF used in our work inherits the same intermolecular interaction terms as the 2004 Gee et al. TATB FF, and thus is expected to yield similar results. However, their value for the (001) facet is 62% lower than we obtain here. It is unclear how to compare our results to the Gee et al. calculation because they used the surface tension approach to compute γ, which would include entropy effects but is now understood to carry an uncharacterized systemic error when applied to solids.^26^

Direct comparisons between the MD values and experiment are more difficult to make. All available experimental measurements of TATB surface energy correspond to values for powders rather than pristine crystal facets. The experimental values exhibit modest spread (≈15 mJ·m^–2^) and are also consistently lower than both others’ and our predicted values for molecularly smooth surfaces. Based on the results of Brahmbhatt and Chaudhuri^29^ mentioned above, it is likely that part of this discrepancy arises from neglecting explicit entropy contributions beyond the effect of averaging over the internal energies of configurations sampled by finite-temperature MD simulations. However, real TATB sample surfaces are undoubtedly more morphologically complicated than the pristine surfaces in MD simulations. Typical production-quality TATB crystals exhibit many defects and substantial surface roughness,^17^ as well as chemical impurities.^82^ The fact that the experimental values are lower than the computed surface energy of the most-stable molecularly smooth TATB facet may indicate that surface roughness plays an important, but still poorly understood role in determining the effective surface energy on larger length scales. The calculated large dispersion component of the TATB surface energy found here is consistent with the contact angle measurements, which indicate the dispersion component being >90%.^10,71–74^ However, this also highlights a qualitative difference among the experiments, as the Wilhelmy plate measurements by Rivera and Matuszak^70^ found roughly 75% of the measured surface energy arises from the polar component. It is unclear whether this discrepancy arises from morphological differences between the various experimental TATB samples or if it is methodological in nature.

Interface energies measured relative to the bulk reveal that each system type exhibits distinctly different magnitudes and physical interaction contributions to the net energetic penalty to form the interface. The most unfavorable interface (relative to bulk) is the TATB(001)-TATB(011) crystal–crystal interface. The crystal–crystal interface energy is dominated by molecular interaction terms, and these contributions are larger for the crystal–crystal interface than for the crystal-polymer interface in both an absolute and relative sense. This indicates that the conformations of molecules at crystal–crystal interfaces are more strongly perturbed than at crystal-polymer interfaces, which matches physical intuition based on the much larger difference in mechanical compliance of the two phases in a crystal-polymer interface. That is, the polymer can more easily strain and adopt an energetically equivalent conformation compared to molecules constrained to lattice sites. We also note the crystal–crystal interface is distinct in that the electrostatic interactions at the interface are energetically favorable compared to the bulk.

In comparison to the crystal–crystal interface, all three interaction types (*E*_VDW_, *E*_Coul_, and *E*_Mol_) are energetically unfavorable for both crystal-polymer interfaces. The two crystal-polymer interfaces exhibit significant differences relative to each other in both the absolute and relative contribution of each interaction type. For instance, the TATB(001)-Ultraflex interface is the least unfavorable (Γ = 32.08_–6.66_^+5.51^ mJ/m^2^), and the majority of this energetic penalty is due to perturbations in molecular conformation rather than intermolecular interactions. In contrast, the TATB(011)-Ultraflex interface exhibits a much larger penalty (74.07_–6.99_^+5.96^ mJ/m^2^), which is due in equal parts to dispersion and electrostatic forces with minimal contribution from intramolecular interactions. Similarly to the free surface case, the larger electrostatic contribution for the (011) case in comparison to the (001) facet is due to the formation of the (011) surface breaking the intermolecular hydrogen-bond network.

The fact that all computed γ and Γ are positive indicates that at the nanometer scale of the modeled interfaces, there is an energetic driver for grain coarsening in TATB-Ultraflex composites. Based on these MD results for the two lowest energy facets we expect this trend to extrapolate in general to high index (higher energy) facets. However, the kinetics for coarsening are almost certainly slow. TATB exhibits very low volatility,^83^ so TATB mass transport via gaseous diffusion is unlikely. Mass transport of Ultraflex is more plausible, given that it has a reported glass transition temperature of −40 °C.^64^ While the present set of MD calculations do indicate an energetic driver exists for grain coarsening in TATB-Ultraflex composites, they do not provide insight into the kinetics of this process.

Real crystals exhibit free surfaces, so the adhesion energy Γ′ provides a better measure to determine whether TATB crystals will adhere to each other and to Ultraflex polymer. As can be seen in Figure 8, all three interface types are energetically favorable. Ultraflex adheres to both TATB facets with similar strength, with −107.68_–7.08_^+5.94^ mJ/m^2^ for the (001) case and −125.47_–8.04_^+15.79^ mJ/m^2^ for the (011) case. Adhesion of Ultraflex to TATB is also mediated almost exclusively by dispersion forces. In contrast, the TATB crystal–crystal adhesion energy is even stronger (−161.73_–2.57_^+2.71^ mJ/m^2^). While TATB crystal–crystal adhesion is still largely due to dispersion forces, there is a significant attractive contribution from electrostatics and repulsive contribution from intramolecular terms. Overall, the predicted Γ′ indicate that TATB crystals exhibit a stronger energetic driver to stick to each other than to a binder made of Ultraflex polymer. Favorable crystal–crystal adhesion is also qualitatively consistent with the wide range of morphologies seen in polycrystalline TATB particles.^17^

### 4.2 Interface Structure

The preceding calculations indicate that TATB exhibits anisotropy in adhesion to Ultraflex polymer, with dispersion, electrostatic, and intramolecular forces each contributing in differing absolute and relative magnitudes to the overall crystal-polymer interaction strength for the (001) and (011) facets. Here we examine the structure of these interfaces to better isolate specific chemical interactions that contribute to TATB-Ultraflex adhesion.

Density profiles ρ­(*z*) of the Ultraflex polymer were computed along the direction normal to the interface for both crystal facet interfaces. Here, ρ­(*z*) was computed by binning the system along *z* with a bin size of Δ*z* = 0.5 Å. Position *z* is measured relative to the interface location, which we define as the minimum in the density profile computed using *both* TATB and Ultraflex atoms. A single trajectory frame was taken from each of the 5 independent simulations used to compute Γ for a given facet case, yielding a total of 10 independent interface configurations. The average density ⟨ρ­(*z*)⟩ was computed over the 10 configurations and the uncertainty was taken to be the 95% confidence interval computed for each bin using *t*-statistics.

Figure 9 shows the computed average Ultraflex density profiles for the TATB(001)-Ultraflex and TATB(011)-Ultraflex interface cases. Both interfaces exhibit pronounced structuring in ρ­(*z*) near the interface, which decays over a distance of approximately 15–20 Å. This indicates that the effective thickness of the interfacial region is quite small compared to the dimensions of Ultraflex polymer chains. In this case, 20 Å corresponds to a contour length of ≈10 monomers and is comparable to the radius of gyration (24 Å) for the 128-mer chains.

**Figure 9:**
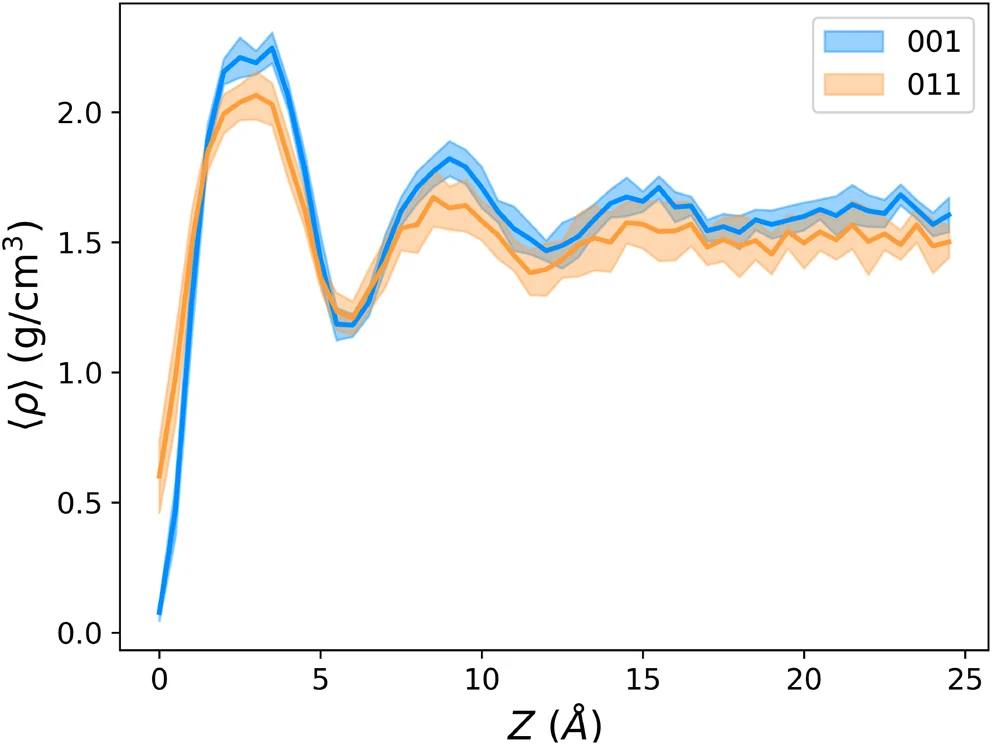
Ultraflex mass density profile ρ­(*z*) along the direction normal to the interface, averaged over all independent interface configurations for both the TATB(001)-Ultraflex case (blue) and the TATB(011)-Ultraflex case (orange). Shaded bands indicate 95% confidence intervals.

Ultraflex is systematically less dense when adhered to the (011) facet compared to the (001) facet. In particular, the first and second density maxima near the (011) interface are respectively reduced by roughly 0.20 g/cm^3^ and 0.15 g/cm^3^ relative to the (001) interface. Smaller, but statistically significant differences in the two profiles extend further into the bulk. Far from the interface, the Ultraflex density profiles approach approximately constant bulk values of 1.60 g/cm^3^ and 1.51 g/cm^3^ for (001) and (011) cases, respectively. The realized bulk densities are somewhat smaller than the value of 1.68 g/cm^3^ we obtained from quenching the pure polymer melt to (300 K, 1 atm) using *NPT* dynamics at a rate of 20 K/ns. Differences in the realized bulk density are reflected in the bulk energy per Ultraflex chain shown in Figure 7, panels (a) and (e). We note that the regression approach to compute Γ automatically accounts for these differences in the realized bulk density and its impact on bulk energy. Such functional dependencies would need to be precisely and extensively mapped in order to accurately apply the direct Γ calculation approach.

These differences in density between the TATB(001)-Ultraflex and TATB(011)-Ultraflex interface cases arise despite the fact that both interface types were constructed using identical protocols. Thus, it is plausible that differences in adhesion energy and interfacial polymer density arise due to anisotropy in the topology and chemistry of these two TATB crystal facets. For instance, the topology of the TATB (001) facet is considerably smoother at an atomic level compared to (011). Atomic-scale roughness of the (011) facet may present a surface with more local voids that hinders close packing of chains and leads to a lower density for the TATB(011)-Ultraflex interface. Additionally, the higher density of Ultraflex at the interface with (001) implies more atom–atom pairwise interactions and correlates positively with the smaller, more energetically favorable value of Γ that was computed in Section 4.1.

To further elucidate the effects of crystal facet topology and chemistry at the interface, we define and compute an interfacial coordination number for each central TATB atom of type α by summing smoothly weighted^84^ pairwise contacts with Ultraflex atoms β across the interface

16 $$N_{c,\alpha }=\sum_{\beta \in \mathrm{Ultraflex}}\frac{1-{\left(r_{\alpha \beta }/r_{0,\alpha \beta }\right)}^{6}}{1-{\left(r_{\alpha \beta }/r_{0,\alpha \beta }\right)}^{12}}$$

Here, *r*_αβ_ is the distance between the central TATB atom α and an Ultraflex atom β across the interface. The characteristic distance *r*_0,αβ_ is defined for each atom pair as the sum of their van der Waals radii, which are *r*_C_ = 1.70 Å, *r*_H_ = 1.20 Å, *r*_N_ = 1.55 Å, *r*_O_ = 1.52 Å and *r*_F_ = 1.35 Å. This pair-dependent characteristic distance accounts for differences in atomic size between chemical species, and the summation gives the total coordination number associated with each central TATB atom. Any given TATB atom is considered to be an “interface atom” if it has at least one Ultraflex atom within a distance of *r*_cut_ = 6.0 Å (and vice versa). All coordination number calculations were performed using instantaneous interface configurations sampled at the very end of the *NVT* simulations used to compute Γ. Average values ⟨*N*_c_⟩ and uncertainties were determined by averaging over the five independent crystal-polymer interface simulations performed for the (001) and (011) cases. Coordination numbers are compared between the (001) and (011) facets by normalizing ⟨*N*_c_⟩ with respect to the number of molecules *N*_TATB_ in the single crystal layer closest to the interface. These values are respectively 224 and 280 for the (001) and (011) facets. Thus defined, our calculations yield the average number of contacts of a given type per TATB molecule at the interface.

Figure 10 shows snapshots of each TATB-Ultraflex interface type, including a side view of the interface, the TATB surface that is in contact with the Ultraflex, and the interfacial Ultraflex atoms identified using the above coordination number analysis for two representative systems. Meshes showing regions where the TATB surface is covered by the polymer atoms were computed using the α-shape method implemented in OVITO.^48^ These meshes were determined using a probe radius of 3.4 Å, which corresponds to the van der Waals diameter of a carbon atom. Thus, void regions identified with the surface mesh are locations where one or more additional atoms could plausibly be inserted.

**Figure 10:**
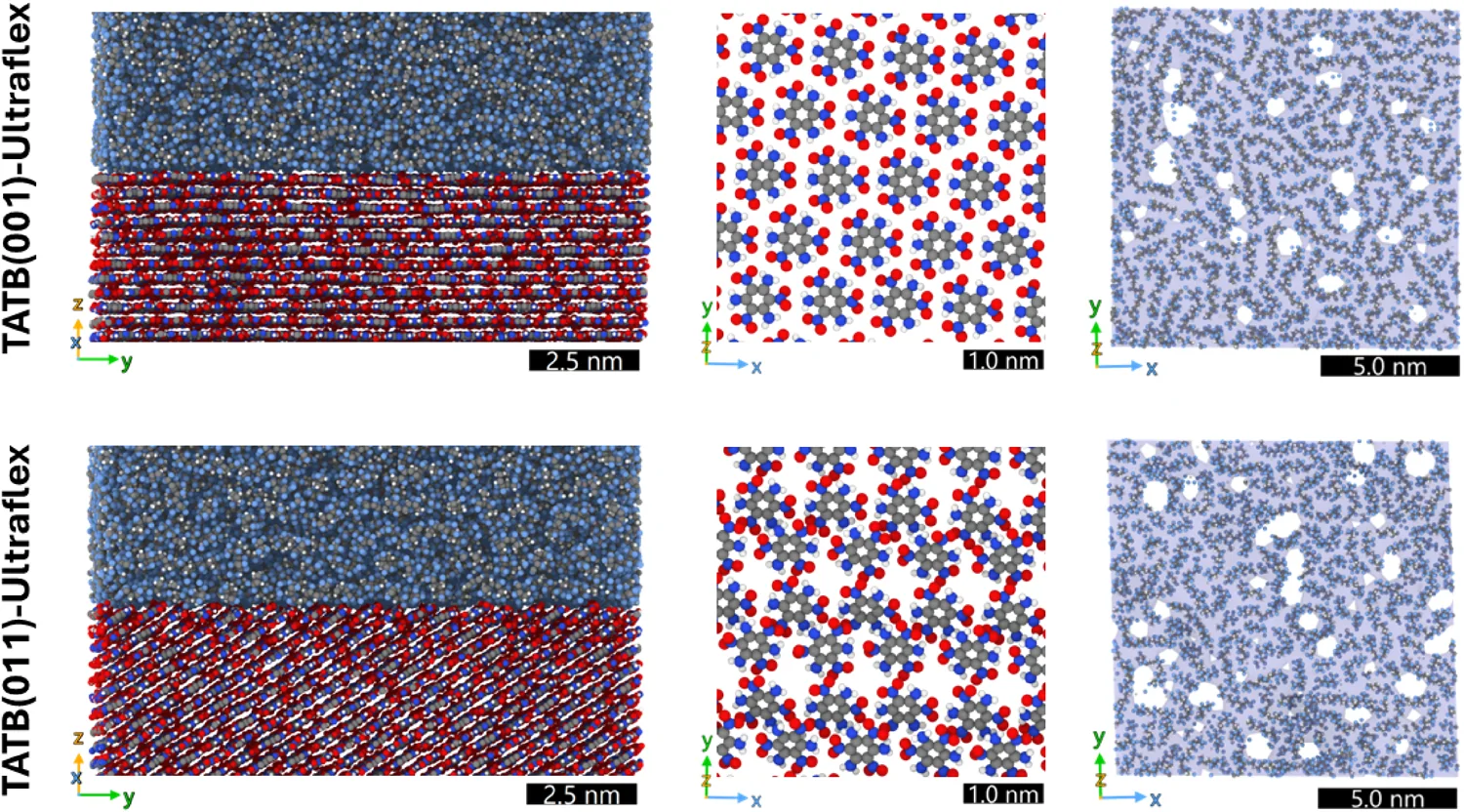
First column: Side view (*y*-*z* plane) of the interface formed between Ultraflex and TATB (001) and (011) facets. Second column: Top view (*x*-*y* plane) of the single crystal TATB layer that is closest to the interface. Third column: Top view (*x*-*y* plane) of the interfacial Ultraflex atoms in contact with the TATB (001) and (011) facets. A semitransparent surface mesh is rendered to show the continuous connected region of polymer demonstrating small voids where the polymer does not cover the TATB surface. Atoms are colored white, gray, dark blue, red, and light blue for H, C, N, O, and F.

Both TATB facets exhibit qualitatively similar structuring of the polymer within the plane of the interface. In particular, we note that neither facet is perfectly wetted by the polymer. Rather, the interfacial region exhibits nanoscopic voids that are on the order of 1 nm in diameter. These voids are roughly an order of magnitude smaller than typical void sizes measured in TATB formulations prepared with fluoropolymers related to Ultraflex,^8^ but this type of feature could plausibly contribute to the tails of measured void-size distributions. We also observe that long contiguous segments of the 128-mer Ultraflex chains adhere to the TATB surface, as opposed to chain terminations.

Figure 11 shows the average coordination numbers for each facet type and for each TATB-Ultraflex atom pair type. The TATB(001)-Ultraflex interface exhibits a higher degree of coordination than the TATB(011)-Ultraflex interface across all pair types. This difference is especially pronounced (in a relative sense) for contacts involving the TATB ring carbon atoms C_TATB_ (blue bars). Here, the TATB(001) facet has nearly double the number of contacts compared to the TATB(011) facet. Greater overall polymer coordination to the (001) facet can be rationalized in terms of sterics of the crystal surface molecules. The (001) surface is an atomically smooth plane that affords essentially equal access to all TATB chemical moieties. That is, there is no steric shielding on one side of each planar TATB molecule at the surface. In contrast, the (011) facet surface molecules are tilted by approximately 32° with respect to the interface plane, and these molecules partially overlap in a manner akin to roofing shingles. This “shingling” sterically shields some of the TATB nitro and amine groups, preventing them from closely coordinating to polymer atoms.

**Figure 11:**
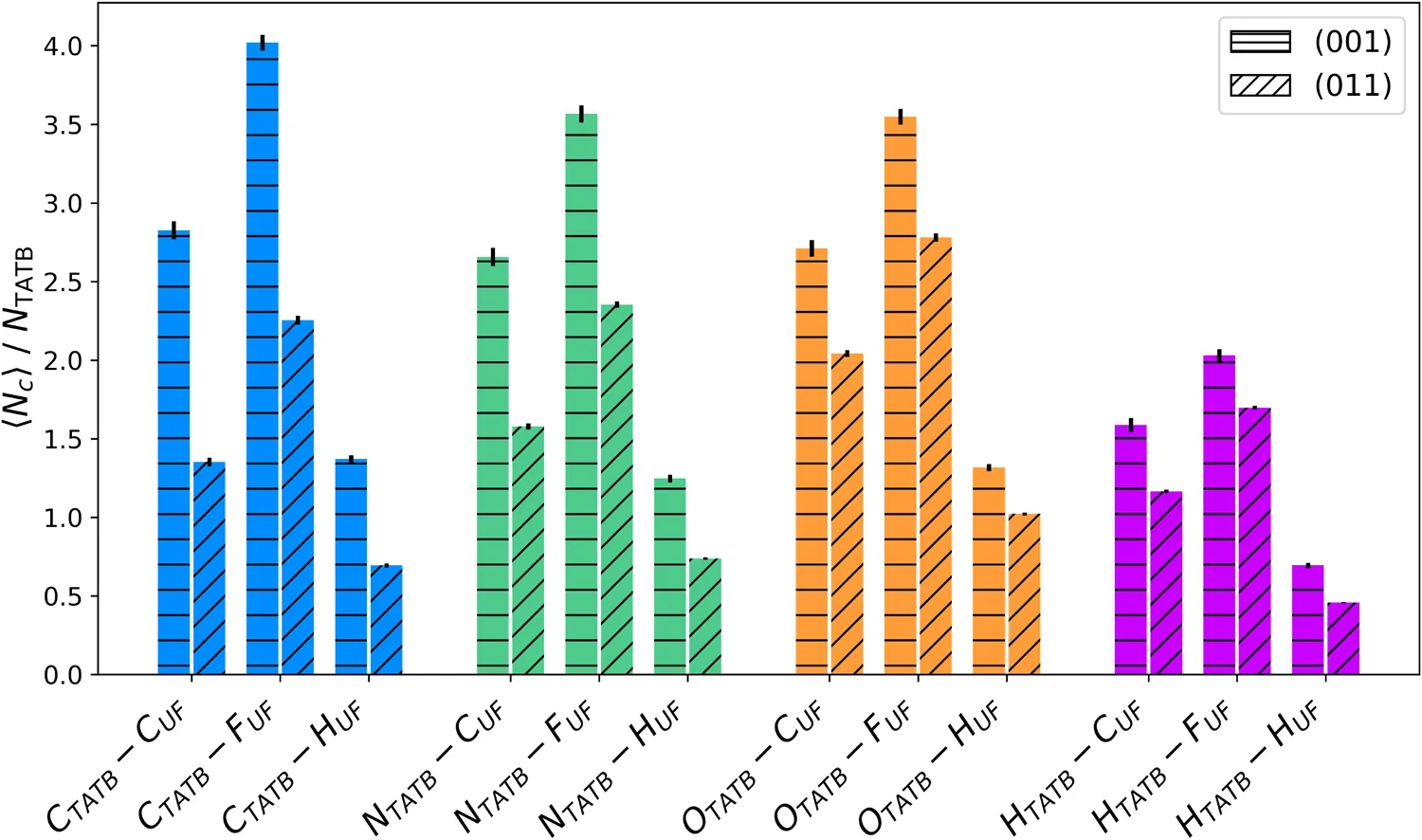
Average coordination number per TATB molecule for pairs of interfacial TATB and Ultraflex atoms for each unique pair of element types. Horizontal hatched bars correspond to the (001) facet and diagonal hatched bars to the (011) facet. Color is used to indicate TATB atom element type, with blue, green, orange, and purple respectively denoted TATB carbon, nitrogen, oxygen, and hydrogen.

Several chemical trends are apparent in the computed coordination numbers. First, the relative coordination of TATB atoms to each element type in Ultraflex always ranks as *N*_c_^H^ < *N*_c_^C^ < *N*_c_^F^. This is consistent with the different number densities for each element type in Ultraflex, which exhibits a H:C:F ratio of 1.0:1.6:2.3. TATB has a molecular formula of C_6_N_6_O_6_H_6_, so any inequalities in the coordination number of TATB atoms to a fixed Ultraflex atom type indicate preferential formation of specific atom–atom contacts. For example, in the (001) case, carbon in Ultraflex (C_UF_) exhibits roughly equal coordination numbers for TATB carbon, nitrogen, and oxygen atoms, but TATB hydrogen is comparatively undercoordinated. Comparing both facets, Ultraflex is generally most highly coordinated to TATB carbon atoms (blue bars), roughly equally coordinated to TATB nitrogen and oxygen atoms (green and orange bars), and least coordinated to TATB hydrogen atoms (purple bars).

The above finding, namely that the smoother TATB (001) facet exhibits a larger overall degree of contact with Ultraflex than the rougher (011) facet, is perhaps counterintuitive. Geometrically, a rougher surface would be expected to have greater effective surface area available to form close contacts with the polymer. Indeed, experimental work by Yang et al.^85^ showed that surface wetting for the related explosive LLM-105 could be improved by an acid etching treatment, although the length scale for this imparted roughness was not quantified. From the data at hand, enhancements to atom–atom close contacts at the crystal-polymer interface do not necessarily translate to stronger, more favorable adhesion energies. However, the number of cases considered here (two crystal facets and one polymer) are too sparse to infer general structure–property relationships.

## 5 Conclusions

PBXs are morphologically complex systems in which the adhesive interactions between the polymeric binder and energetic molecular crystals are thought to influence functional properties including mechanical strength, safety, performance, and aging characteristics. However, it remains difficult to directly measure adhesive interactions through experiments. Such information is largely inferred from surface energy measurements, which can be highly sensitive to both measurement methodology and sample preparation. The microscale features that govern crystal-polymer adhesion are difficult to resolve experimentally, but can be readily accessed through all-atom modeling techniques such as MD.

To this end, we extend MD methods to accurately predict the cohesive properties of interfaces in a prototypical explosive comprised of the insensitive HE TATB and the fluoropolymer Ultraflex. TATB exhibits what is arguably the greatest intrinsic anisotropy of any HE in common use due to its layered, low-symmetry triclinic crystal structure. This makes TATB ideal for a case study, both because methods that accommodate its triclinic structure must be fully general and because its intrinsic anisotropy can possibly exaggerate anisotropic effects at interfaces. The all-atom MD simulations performed here depend on three core elements: (1) an accurate classical all-atom MD FF for the TATB-Ultraflex composite; (2) general simulation cell construction protocols to model crystal-polymer and crystal–crystal interfaces; and (3) a numerically robust approach to compute the surface (γ) and interface (Γ) energy from MD-derived energies of surface and interface systems.

All MD simulations were performed using a composite FF that combines the CLM FF for TATB with the general-purpose polymer model PCFF+. The FF description of the intermolecular potential energy surface governing TATB-Ultraflex interactions was validated against DFT calculations and exhibits errors that are less than *k*_B_ *T* at room temperature. Construction protocols to build MD simulation cells of oriented crystal–crystal and crystal-polymer interfaces were developed that build upon the GCCM. In particular, we developed a multistep MD workflow that mimics HE pressing operations to construct 2D periodically infinite crystal-polymer interfaces.

It is well understood that directly computing the surface energy as a difference between the energy of a surface system and bulk system is numerically unstable. A formal analysis of the condition number for this calculation shows that this approach is ill-suited to the large system sizes needed when modeling polymers. In this respect, we adopt an established regression-based approach to compute the surface energy and extend it to compute interface energy. This approach adds computational cost in the sense that multiple independent simulations of an interface must be performed, but it avoids poor numerical conditioning.

Application of the above MD models and techniques to two idealized, defect-free TATB crystal facets and their interfaces with Ultraflex reveals that surfaces and interfaces in this system are largely dominated by dispersion forces. TATB crystal free surfaces are significantly more energetically unfavorable than Ultraflex surfaces (γ_TATB_ ≥ 3.5γ_UF_) and the TATB surface energies are also highly anisotropic. Predicted interface energies show that crystal–crystal and crystal-polymer interfaces are energetically unfavorable compared to the bulk, indicating that at the nanometer scale of a TATB-Ultraflex PBX there is an energetic driver for grain coarsening. In contrast, predicted adhesion energies are favorable and reveal that while TATB crystals will readily adhere to Ultraflex, they bind even more strongly to each other. These data indicate that the thermodynamic landscape governing formulation design and aging of polymer bonded explosives is complicated and involves additional variables besides crystal-polymer adhesion.

Analysis of TATB-Ultraflex interfaces reveals that the crystal imparts significant structure to the polymer that varies depending on the wetted facet type. The lowest-energy TATB facet, namely the (001) basal facet, promotes enhanced densification of Ultraflex at the interface that is correlated to lower (and thus more favorable) interface energy. However, the spatial scale of the interfacial region is nanoscopic, exhibiting nonbulk-like features in density that are localized to within ≈2 nm of the crystal surface. Nanoscopic voids approximately 1 nm in diameter are also found to spontaneously form at the crystal-polymer interface, which could plausibly contribute to the tails of experimentally measured pore size distributions in TATB-based explosives. Coordination analysis reveals that the crystal facet morphology impacts the average number of TATB-Ultraflex close contacts and that Ultraflex preferentially coordinates to the carbon atoms in the TATB benzene ring.

The interface-construction workflow and regression-based interface energy analysis presented here provide a general strategy for computing surface and interface energies that underlie polymer-crystal composites using MD. This workflow can also be extended to investigate how surface roughness, defects, and temperature affect surface and interface energies. The present results establish a baseline understanding of how modeled TATB crystal facets influence adhesive interactions with Ultraflex and highlight dispersion-dominated, morphology-controlled adhesion as a defining feature of these systems. Open questions remain regarding how crystal-polymer adhesion impacts the material properties of PBXs. In particular, it is unclear if there exist structure–property relationships that relate adhesion energy to the polymer chemistry, polymer molecular weight, and HE crystal facet roughness/chemistry. The influence of adhesion on macroscopic properties such as PBX mechanical strength also remains poorly quantified. However, the MD methods developed here provide a route to supplement this data-poor application area and hold promise as a tool that could aid the design of PBXs with enhanced properties. While the present study focused on explosives, the methodology is general and can be readily adapted to other material systems including organic electronics, pharmaceuticals, and the engineering of advanced functional materials and polymer composites.

## Supplementary Material



## Notes

Bulk polymer chain dynamics, selecting statistically independent data points, comparing interface energy results with an NPT ensemble versus NVT ensemble, block size selection for interface energy and surface energy calculations and tabulated surface and interface energies (PDF)
