# Supplementary material for: Predicted Cohesive Properties of a TATB Polymer-Bonded Insensitive High Explosive
Source: J Phys Chem C Nanomater Interfaces. 2026 Oct 7;130(42):14660–77. doi: 10.1021/acs.jpcc.6c03771 (PMC13700089; doi:10.1021/acs.jpcc.6c03771)
Supplement: Supplementary file 1 [file jp6c03771_si_001.pdf]

Supporting Information: Predicted Cohesive Properties of a TATB Polymer-Bonded Insensitive High Explosive

Leo W. Zella,* Nicholas T. Liesen, H. Keo Springer, and Matthew P. Kroonblawd*

Physical and Life Sciences Directorate, Lawrence Livermore National Laboratory, Livermore, CA 94550, United States

E-mail: zella1@llnl.gov; kroonblawd1@llnl.gov

1 Bulk polymer chain dynamics

Linear polymer chain relaxation dynamics scale with the polymerization degree N (chain length), transitioning from an unentangled, Rouse-like regime for small N to an entangled regime for large N. Consequently, the time required to obtain effectively decorrelated configurations depends on the underlying dynamical regime of the chains. To determine sufficient equilibration time between effectively decorrelated bulk configurations of Ultraflex, we compute the autocorrelation function $C_{\mathrm{EE}}(\tau)$ of the chain end-to-end separation vectors $\mathbf{R}_{\mathrm{EE}}$ as

$$C_{\mathrm{EE}}(\tau) = \frac{\langle \mathbf{R}_{\mathrm{EE}}(\tau) \cdot \mathbf{R}_{\mathrm{EE}}(0) \rangle}{\langle R_{\mathrm{EE}}^2(0) \rangle} \approx \exp\left[-\left(\frac{\tau}{\tau_{\mathrm{EE}}} \right)^{\beta} \right], \quad (1)$$

where τ_{EE} is an effective decorrelation time and β is a stretching exponent that characterizes the underlying relaxation dynamics.

We assessed $\tau_{\mathrm{EE}}(N)$ for monodisperse Ultraflex polymer melts at 600 K and 1 atm using NPT MD simulations for degrees of polymerization $N = 8, 16, 32, 64$, and 128. Bulk polymer configurations were prepared following the protocol described in the main article. To obtain adequate statistics for the end-to-end correlations, ensembles of simulations were performed with 10 independent simulations for the $N = 8, 16$, and 32 systems, and 5 simulations for the $N = 64$ and 128 systems. For each trajectory, $C_{\mathrm{EE}}(\tau)$ was computed and the average $\langle C_{\mathrm{EE}}(\tau) \rangle$ was computed using bootstrap resampling with 500 draws. The averaged autocorrelation was then fit by least squares to the stretched exponential form in Equation 1.

Figure S1 shows $C_{\mathrm{EE}}(\tau; N)$ and $\tau_{\mathrm{EE}}(N)$ for the five chain lengths considered. Fitting $\langle C_{\mathrm{EE}}(\tau) \rangle$ to Equation 1 yields a set of relaxation times τ_{EE} and stretching exponents β, which are tabulated in Table S1. From this set of relaxation times, a fit to a power law equation of the form $\tau_{\mathrm{EE}} = aN^x$ is utilized to assess the polymer dynamics regime. The fit, shown in panel (b) of Figure S1, yields a scaling exponent of $x = 2.6$. The observed scaling exponent indicates that the polymer dynamics lie in a crossover regime between Rouse-like dynamics, for which the relaxation time scales as $\tau \sim N^2$, and entanglement-dominated

dynamics, which are predicted to scale as $\tau \sim N^3$ in the reptation model.[S1]

Table S1: Dependence of the relaxation time τ_{EE} and stretching exponent β on the degree of polymerization N.

N	τ_{EE} (ns)	β
8	0.016	0.864
16	0.12	0.861
32	0.75	0.874
64	3.41	0.824
128	15.06	0.751

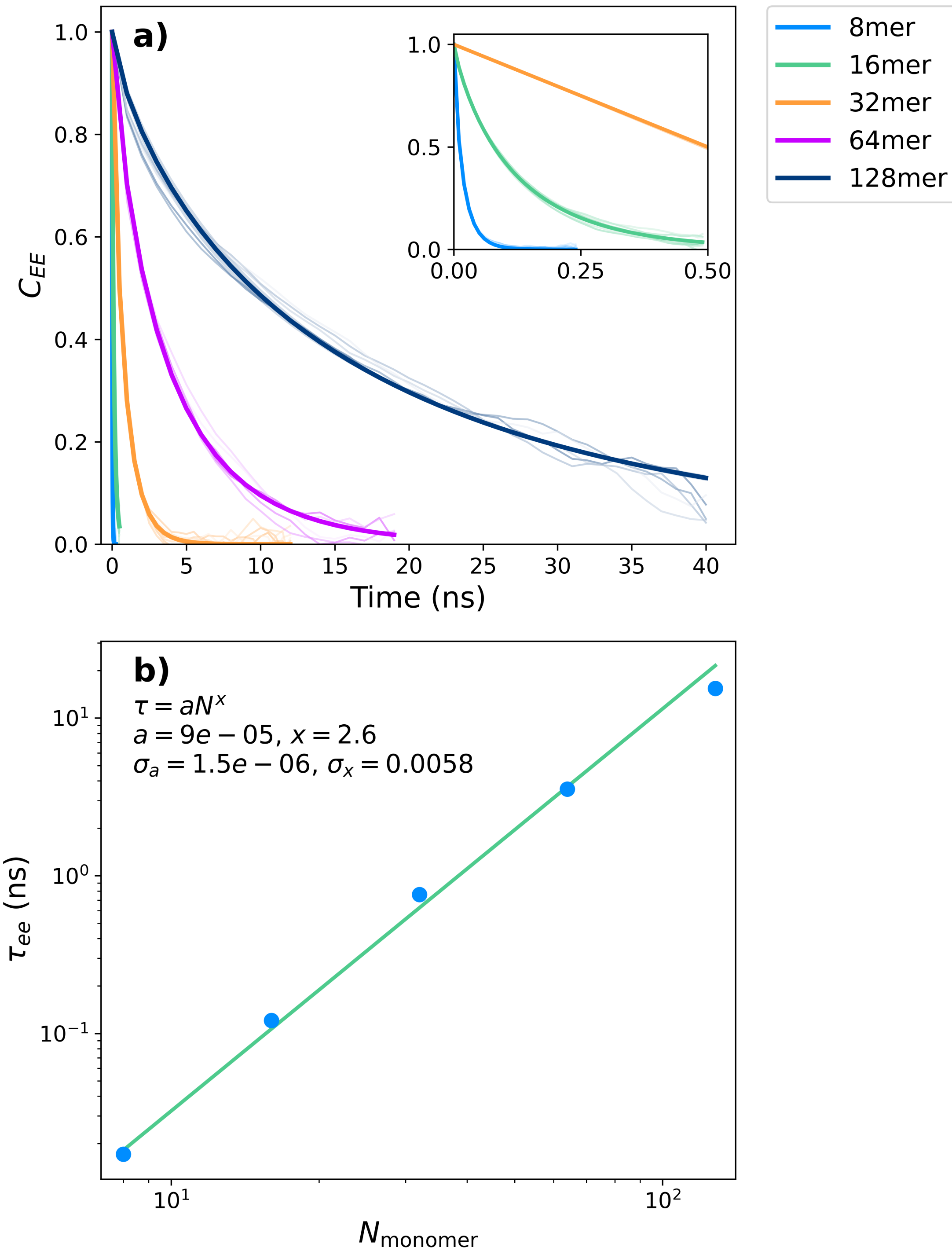

Figure S1: (a) Autocorrelation functions $C_{\mathrm{EE}}(\tau)$ for the chain end-to-end vector $\mathbf{R}_{\mathrm{EE}}$ predicted for Ultraflex polymer melts at (600 K, 1 atm) with $N = 8$, 16, 32, 64, and 128. Solid dark curves correspond to stretched exponential functions fitted to the average $\langle C_{\mathrm{EE}}(\tau)\rangle$ determined from MD. (b) Correlation time τ_{EE} (blue symbols) and power-law fit (green line).

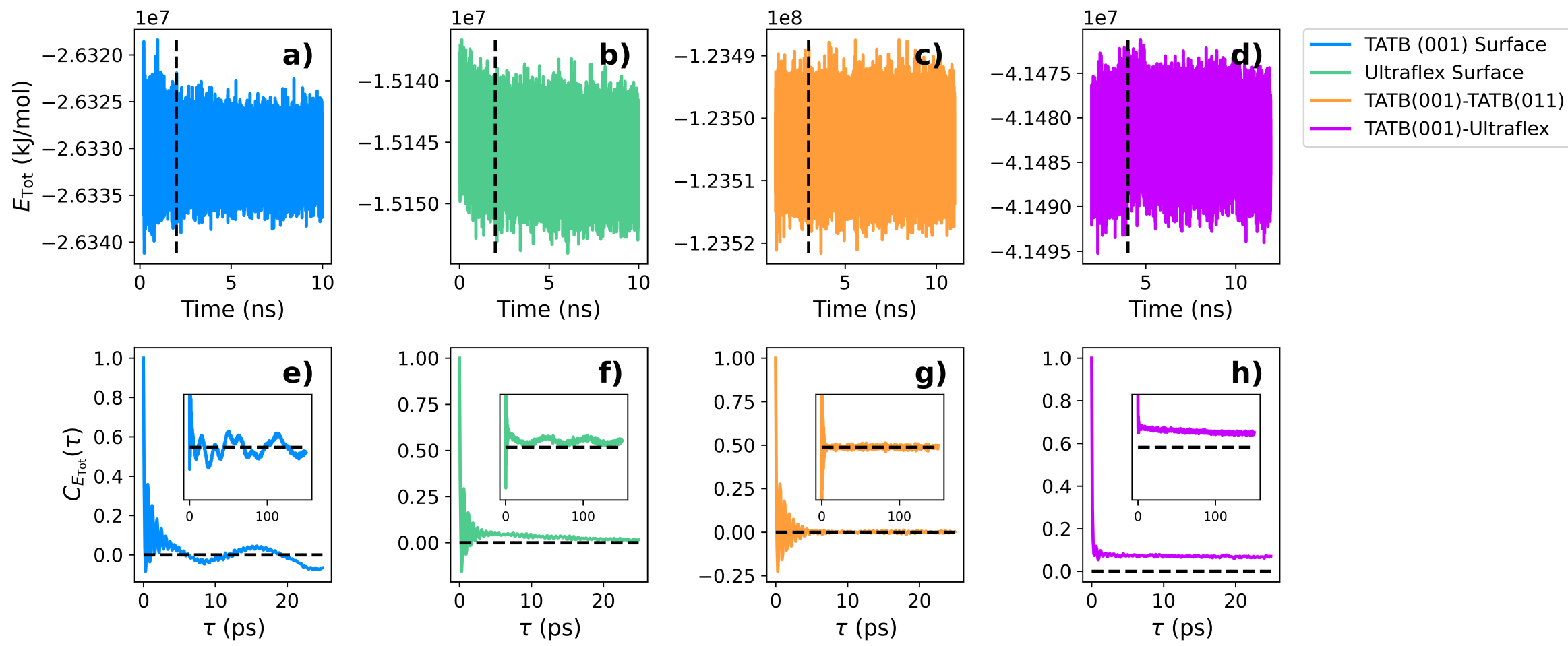

Figure S2: Potential energy and autocorrelation of the potential energy for a TATB(001) free surface, Ultraflex free surface, TATB(001)-TATB(011) interface under an NVT ensemble and a TATB(001)-Ultraflex interface under a NPT ensemble. All simulations utilize a Nosé-Hoover thermostat. Top row is the potential energy for (a) TATB(001) free surface (b) Ultraflex free surface (c) TATB(001)-TATB(011) interface (d) TATB(001)-Ultraflex with a dashed vertical line that indicates the timepoint from which the autocorrelation was calculated. Bottom row is the autocorrelation of the potential energy, $C_{E_{\mathrm{Tot}}}$ for (e) TATB(001) free surface (f) Ultraflex free surface (g) TATB(001)-TATB(011) interface (h) TATB(001)-Ultraflex interface.

2 Selecting statistically independent data points

Correlations in stress $\sigma_{ij}(t)$ and energy $E(t)$ time series arise in MD simulations due to fundamental material physics (e.g., anharmonic vibrational coupling, wave interactions with microstructure defects) and the choice of dynamical equations of motion (e.g., thermostats). The correlation time τ in a signal, such as the total potential energy $E_{\mathrm{Tot}}(t)$, can be computed from an MD simulation as

$$C_{E_{\mathrm{Tot}}}(\tau) = \frac{\langle (E_{\mathrm{Tot}}(t+\tau) - \langle E_{\mathrm{Tot}} \rangle)(E_{\mathrm{Tot}}(t) - \langle E_{\mathrm{Tot}} \rangle) \rangle}{\left\langle (E_{\mathrm{Tot}}(t) - \langle E_{\mathrm{Tot}} \rangle)^2 \right\rangle}. \tag{2}$$

This effectively sets the minimum sampling period needed to obtain statistically independent measurements of that signal. That is, for a given trajectory length, the number of effectively independent samples n_{eff} is set by the correlation time τ.

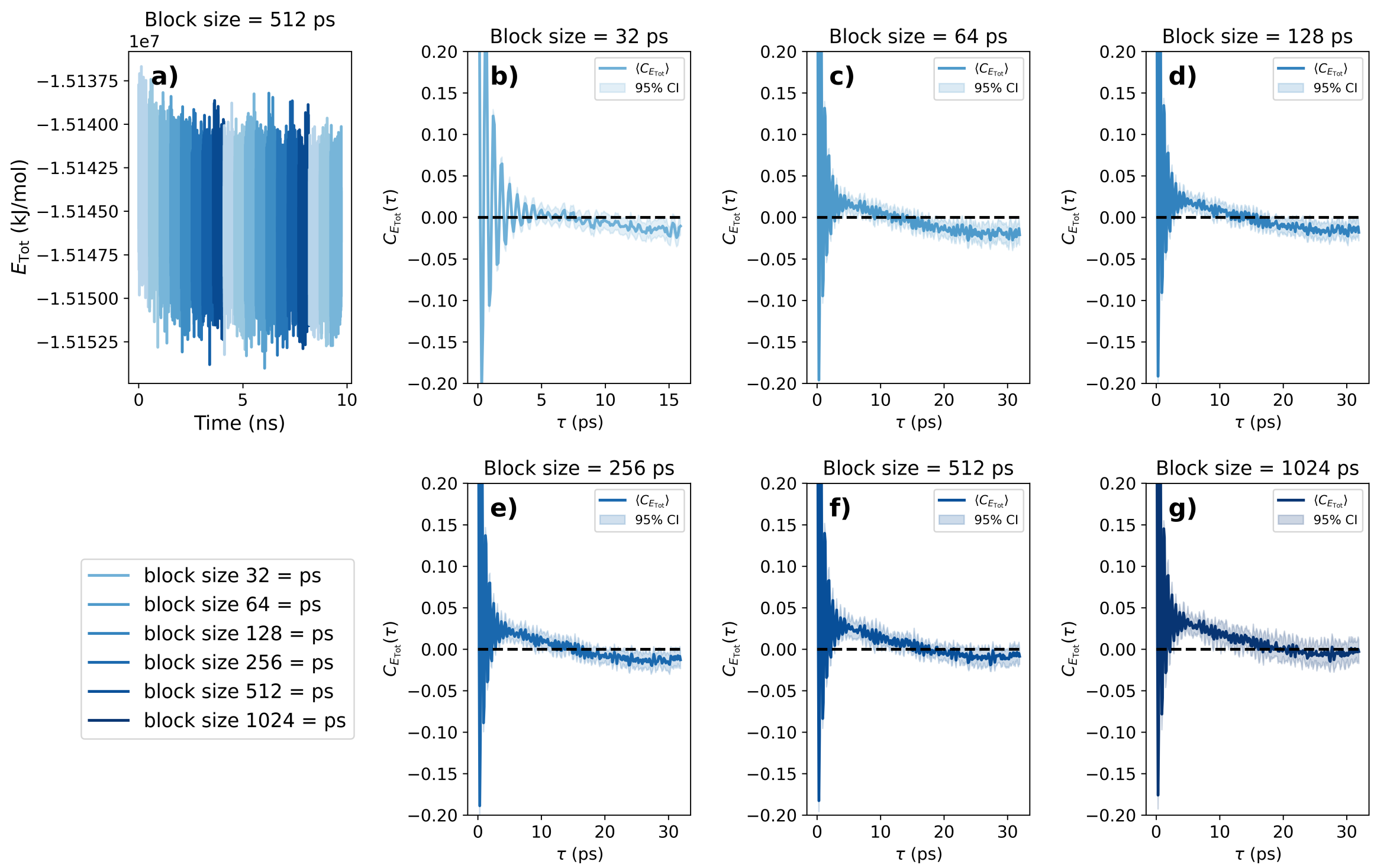

Figure S3: Blocked potential energy and averaged autocorrelation of the potential energy over a variety of blocking sizes for a Ultraflex free surface. The simulation is under an NVT ensemble utilizing an Nosé-Hoover thermostat. (a) Blocked Potential energy, E_{Tot}, with hues differentiating blocks. Averaged autocorrelation $\langle C_{E_{\mathrm{Tot}}} \rangle_{\mathrm{block}}$ for a block size of (b) 32 ps (c) 64 ps (d) 128 ps (e) 256 ps (f) 512 ps (g) 1024 ps. Uncertainty bands represent a 95% confidence interval.

Figure S2 shows the potential energy and autocorrelation of an Ultraflex free surface, TATB(001) free surface, TATB(001)-TATB(011) interface, and TATB(001)-Ultraflex interface. The free surfaces and the TATB(001)-TATB(011) simulations were performed under a NVT ensemble and the TATB(001)-Ultraflex system under a NPT ensemble with all simulations utilizing a Nosé-Hoover thermostat. Autocorrelations were computed after an equilibration time of 2 ns. The autocorrelation functions exhibit long-time features such as long-lived oscillations for the TATB(001) free surface, as seen in panel (e). Similarly, the Ultraflex free surface displays a long-lived oscillation, as seen in panel (f). The TATB(001)-TATB(011) system shows a relatively rapid decorrelation that is stable over time; however, the TATB(001)-Ultraflex interface shows a small constant offset, as seen in panel (h). The

oscillatory behavior found in the autocorrelation of the potential energy for both free surfaces may be due to long-wavelength surface oscillation modes. These long-time correlations reduce the number of effectively independent samples and complicate uncertainty quantification from straightforward time averaging.

To further investigate the long-lived correlations while accounting for a non-stationary signal, we examine the autocorrelation in subdivisions of the time series. The previous analysis computed $C_{E_{\text{Tot}}}$ over the entire time series, which exhibits a systematic drift. Here, we compute the autocorrelation in short pseudo-stationary windows by subdividing the time series into N_{block} non-overlapping blocks. For the ith block, we compute $C_{E_{\text{Tot}},i}$ and average over all blocks to obtain

$$\langle C_{E_{\text{Tot}}} \rangle_{\text{block}} = \frac{1}{N_{\text{block}}} \sum_{i=1}^{N_{\text{block}}} C_{E_{\text{Tot}},i} \tag{3}$$

where N_{block} is the number of blocks and the pointwise standard error for each lag time is computed from the distribution of autocorrelation functions allowing for calculation of uncertainty bands which are chosen at a 95% confidence interval. Figure S3 shows the impact of blocking of a variety of sizes on $C_{E_{\text{Tot}}}$, demonstrating that for all block sizes the autocorrelation decays rather than showing a constant offset as in Figure S2 panel (h). This provides strong numerical evidence that the constant offset arises from the slow drift in the energy time series, which biases the normalization factor in the autocorrelation estimates when a single global mean is used.

Next we examine how oscillatory correlation in the potential energy of the free surface of TATB crystal may be mitigated. A Langevin thermostat can damp long-lived oscillatory correlations, but is sensitive to the choice for the damping parameter, τ_{damp}. The Langevin thermostat[S2] evaluates forces as follows:

$$\mathbf{F}_i(t) = -\nabla_{\mathbf{r}_i} U(\{\mathbf{r}\}) - \frac{m_i}{\tau_{\text{damp}}} \mathbf{v}_i(t) + \sqrt{\frac{2 m_i k_{\text{B}} T}{\tau_{\text{damp}}}}\, \boldsymbol{\eta}_i(t), \tag{4}$$

where $U(\{\mathbf{r}\})$ is the interatomic potential energy, $\mathbf{r}$ is the set of all atomic positions, $\mathbf{v}_i(t)$ is

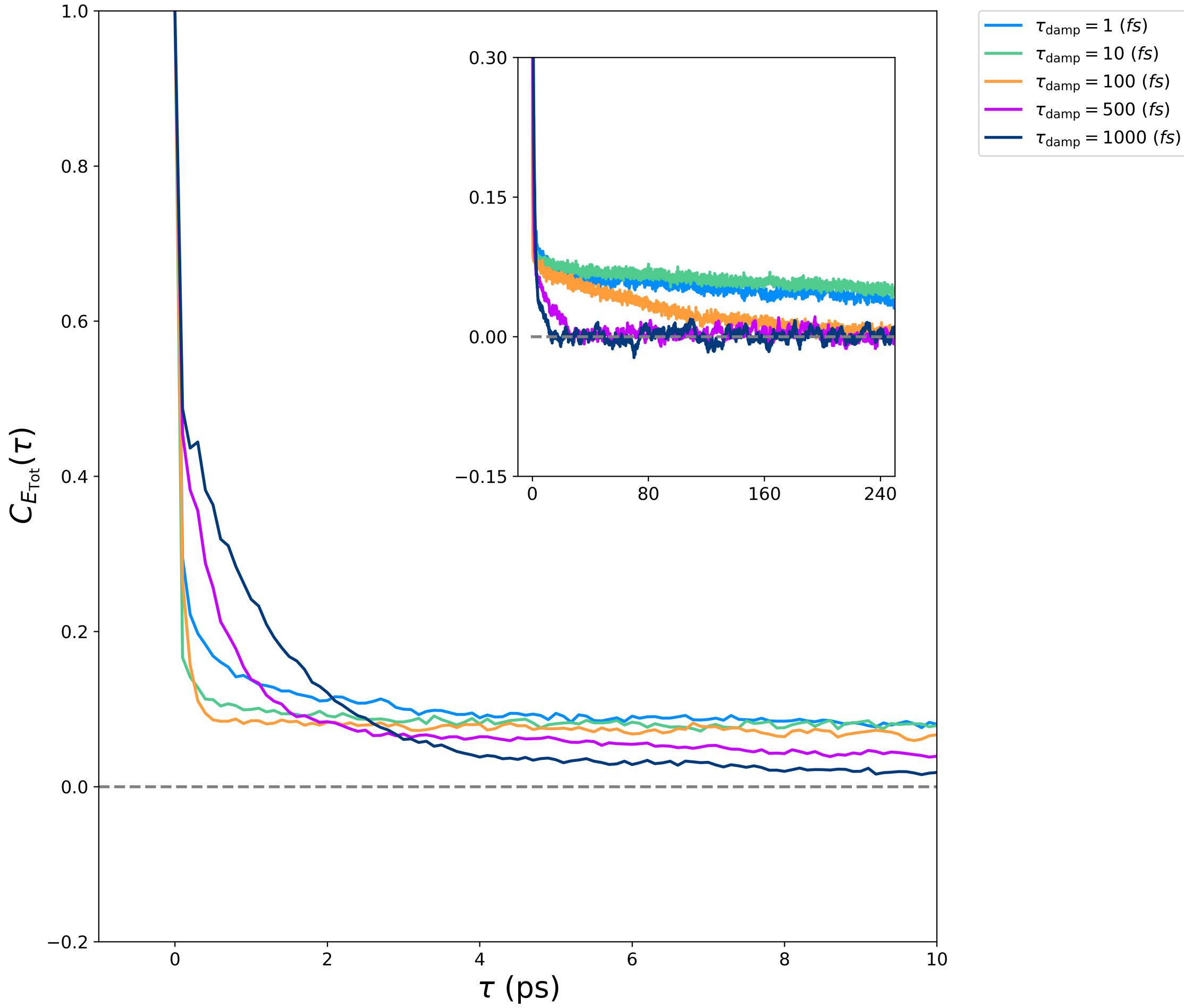

Figure S4: Autocorrelation of the potential energy for a TATB(001) free surface. Simulation is under a NVT ensemble with a Langevin thermostat under a range of different damping parameters.

the velocity of atom i, m_i is the atomic mass of atom i, T is the temperature, τ_{damp} is the damping parameter, and $\boldsymbol{\eta}_i(t)$ is Gaussian random noise with zero mean and unit variance. The additional force terms are a frictional drag term and a random force, which can be tuned to rapidly decorrelate signals.

Figure S4 shows a parametric evaluation of τ_{damp} terms for NVT simulations of a TATB(001) free surface. We see that the short time correlations are most effectively damped using a τ_{damp} of 100 fs with both smaller and larger values taking longer to decorrelate. The downside of this selection is that subtle long-time correlations remain due to the Langevin thermostat increasing the effective viscosity of the dynamics relative to larger τ_{damp}. From

this we select τ_{damp} of 100 fs as the best compromise and note that this parameter does not substantially impact the calculations of the interfacial energy, as discussed in the following section of the Supporting Information.

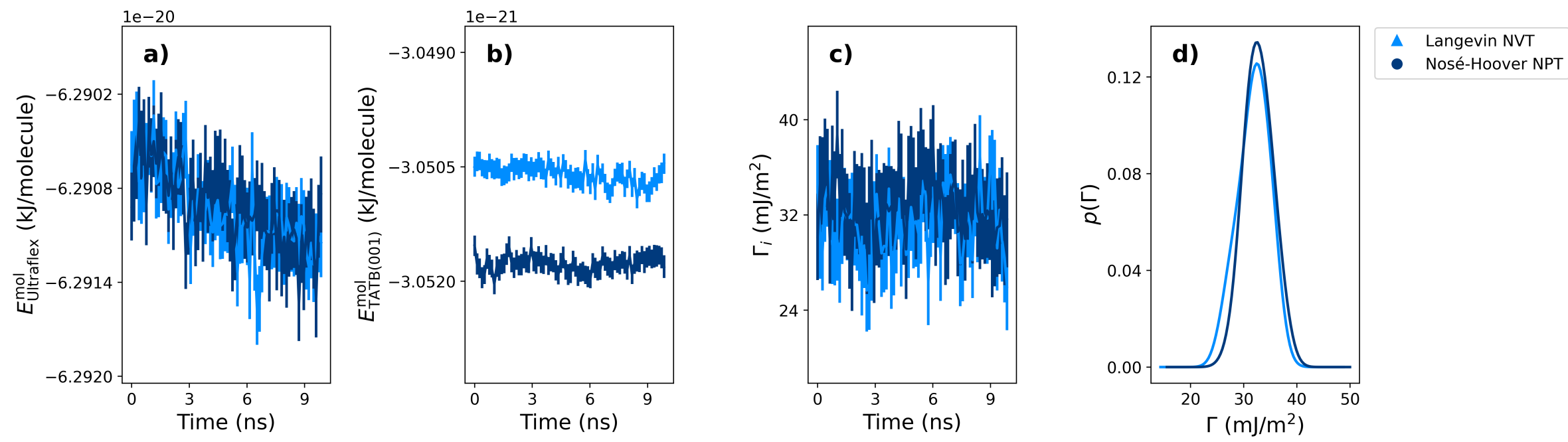

Figure S5: Impact of dynamical equations of motion (NVT vs. NPT) on a TATB(001)-Ultraflex interface, showing time histories for (a) the bulk energy of Ultraflex $E^{\mathrm{mol}}_{\mathrm{Ultraflex}}$, (b) the bulk energy of TATB $E^{\mathrm{mol}}_{\mathrm{TATB}}$, and (c) the interface energy Γ. (d) The sampling distribution $p(\Gamma)$ determined from the last 5 ns of the time history.

3 Comparison of NVT and NPT dynamics for interface energy calculations

The choice of dynamical equations of motion used to sample the energy of crystal-polymer interfaces is nuanced. While NPT-like dynamics with a barostat applied along the cell direction normal to the interface would at first seem the best choice, this is not necessarily the case in practice. The large difference in compliance between the crystal and polymer coupled with the long-lived polymer chain relaxation dynamics leads to non-steady stress/strain conditions depending on whether NVT or NPT dynamics are applied. To evaluate the impact of using NVT or NPT dynamics on the interface energy, we applied a time-series blocking analysis to the TATB(001)-Ultraflex interface system under two different conditions: (i) with NVT dynamics using a Langevin thermostat set to $T = 300$ K with damping time τ_{damp} of 100 fs; and (ii) with NPT-like dynamics at (300 K, 1 atm) with a Nosé-Hoover thermostat and barostat applied along z only.

Figure S5 shows the resulting TATB and Ultraflex bulk energies ($E^{\mathrm{mol}}_{\mathrm{TATB}}$ and $E^{\mathrm{mol}}_{\mathrm{Ultraflex}}$), the interface energy Γ, and the sampling distribution $p(\Gamma)$ determined from the last 5 ns of the 10 ns long trajectories. It is immediately apparent that the predictions for $E^{\mathrm{mol}}_{\mathrm{Ultraflex}}$, Γ, and $p(\Gamma)$ are nearly identical within the fluctuations of the calculation. The one notable

difference arises in $E^{\mathrm{mol}}_{\mathrm{TATB}}$, which is lower under NPT conditions. The higher value for $E^{\mathrm{mol}}_{\mathrm{TATB}}$ under NVT conditions correlates positively with the residual stresses described in the main text. Overall, these results indicate that the residual stresses present in the system do not substantially impact the interface energy.

4 Selecting the block size for interface energy calculations

For the calculation of the time dependence of the interface energy Γ and the surface energy γ, a time-blocking approach has been utilized as described by Equation 14 in Section 3.2 of the main article. Excessively large block sizes t_{block} introduce uncertainty due to the non-stationary potential energy whereas very small t_{block} leads to large uncertainties within each individual block due to the small number of data points from which to compute statistics. We identified an appropriate choice for t_{block} through a parametric study of its impact on $p(\Gamma)$, the sampling distribution for the interface energy. Note that we adopt the same fundamental 2 ps sampling period as described in the main article.

Figure S6 shows the resulting $p(\Gamma)$ computed for the three interface system types using selected t_{block}. For the largest t_{block} size of 512 ps ($N_b = 256$), all three systems display narrow distributions which are quite asymmetric in the case of the TATB(011)-Ultraflex interface and the TATB(001)-TATB(011) interface. The smallest t_{block} size of 64 ps ($N_b = 32$) leads to sampling distributions that are broad and symmetric. Smaller t_{block} were not considered to avoid the added (modest) complication of t-statistics. We identified $t_{\text{block}} = 128$ ps as a reasonable choice across all interface and surface types, since it yielded the smallest 95% confidence interval while retaining an approximately symmetric sampling distribution.

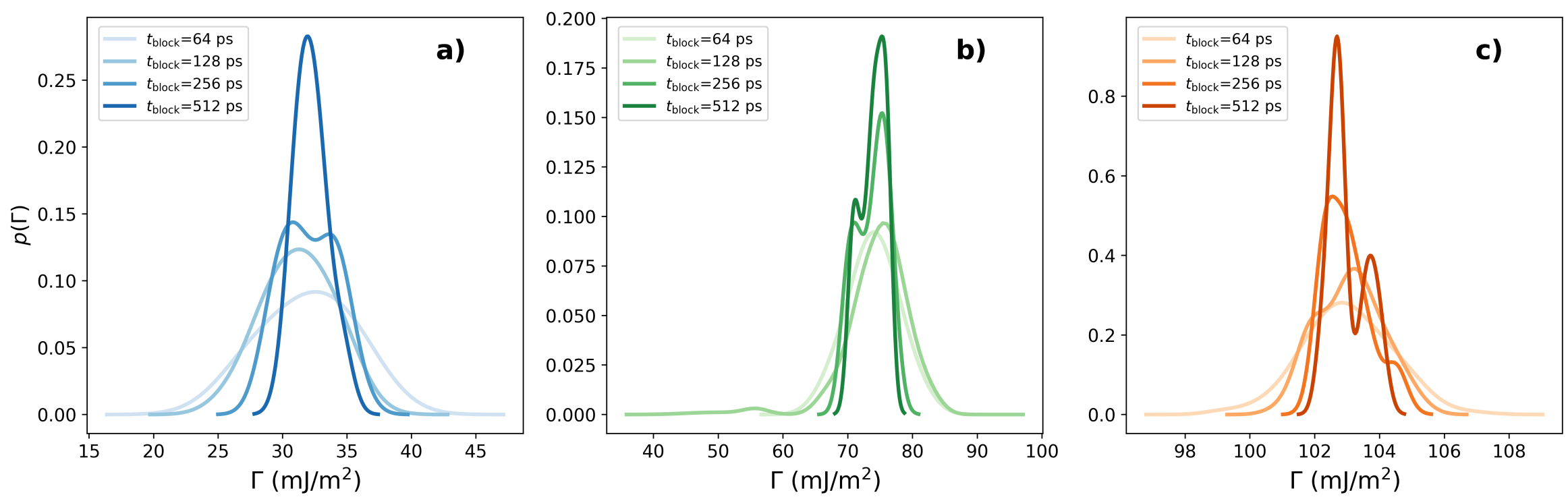

Figure S6: Interface energy sampling distribution $p(\Gamma)$ computed using selected block sizes t_{block} for (a) the TATB(001)-Ultraflex interface, (b) the TATB(011)-Ultraflex interface, and (c) TATB(001)-TATB(011) interface.

5 Surface energy time history

We applied the same time-blocking analysis to surface energy calculations as was described for interface energy calculations in Section 3.2 in the main article. Figure S7 shows these results for all four free surfaces: (i) the free Ultraflex surface obtained by removing TATB from the equilibrated TATB(001)-Ultraflex interface (hereafter denoted Ultraflex(001)); (ii) the analogous free Ultraflex surface obtained from the TATB(011)-Ultraflex interface (Ultraflex(011)); (iii) the TATB(001) free surface; and (iv) the TATB(011) free surface. A block size of 128 ps was applied in all cases and the sampling distributions $p(\gamma)$ were computed using the final 5 ns of the 10 ns long time histories.

We see that for Ultraflex(001), TATB(001), and TATB(011), the γ_i values are stable over time; however, the Ultraflex(011) free surface exhibits large instabilities in the surface energy calculation. These fluctuations in γ_i may be linked to residual stress present in the Ultraflex free surface due to its origin in the interface system. Even with an additional 10 ns relaxation time for the Ultraflex surfaces, the instability was still present in the Ultraflex(011) free surface. This results in a bimodal $p(\gamma)$ distribution (see column 3, row 2), from which we are still able to extract an averaged γ, albeit with more uncertainty than for the Ultraflex(001) case.

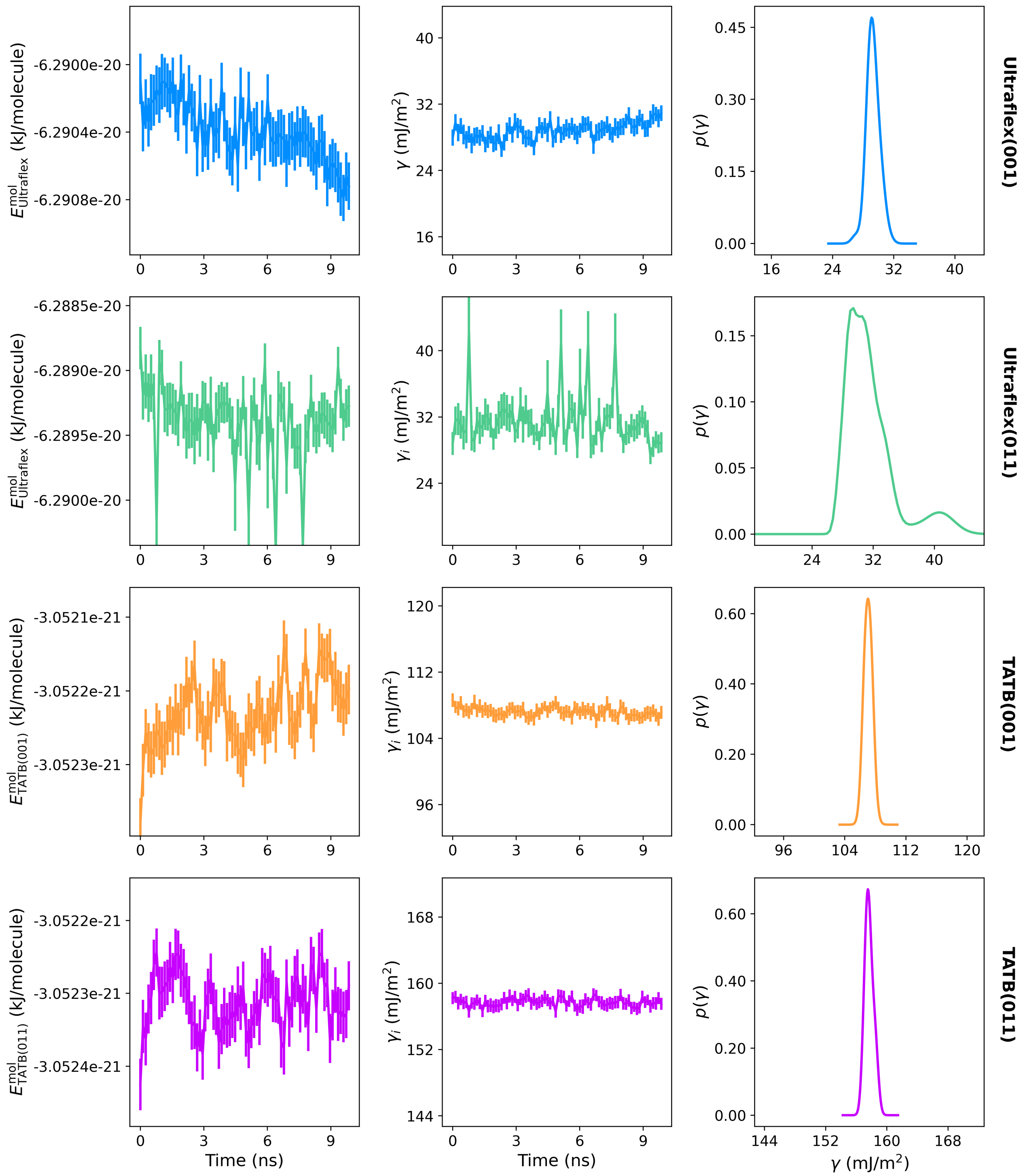

Figure S7: Time histories for the bulk energy and surface energy determined from the regression approach as applied to all four different free surface types (rows). Plots show the bulk energy per molecule (left column), the surface energy (second column), and the sampling distribution for the surface energy $p(\gamma)$ computed from the last 5 ns (right column).

6 Tabulated surface and interface energies

Table S2 gives the tabulated total interface and surface energies for all constructed system types with the separated potential energy contributions due to L-J van der Waals interactions E_{VDW}, Coulomb electrostatics E_{Coul}, and the molecular covalent terms E_{Mol}. Values represent the computed medians, lower uncertainty (2.5th percentile) and upper uncertainty (97.5th percentile) from the sampling distributions of $p(\gamma)$ and $p(\Gamma)$.

Table S2: Interface and surface energies

System	E_{Tot}	E_{VDW}	E_{Mol}	E_{Coul}
TATB(001) Free Surface (γ)	$107.06^{+1.16}_{-1.12}$ mJ/m^2	$87.77^{+0.72}_{-0.68}$ mJ/m^2	$8.15^{+1.02}_{-1.13}$ mJ/m^2	$11.12^{+0.91}_{-0.86}$ mJ/m^2
TATB(011) Free Surface (γ)	$157.65^{+1.31}_{-1.04}$ mJ/m^2	$104.56^{+0.69}_{-0.84}$ mJ/m^2	$10.73^{+1.05}_{-1.1}$ mJ/m^2	$42.45^{+0.93}_{-0.97}$ mJ/m^2
Ultraflex Free Surface (001 interface) (γ)	$32.71^{+1.89}_{-2.14}$ mJ/m^2	$34.54^{+1.44}_{-1.36}$ mJ/m^2	$1.16^{+1.58}_{-1.52}$ mJ/m^2	$-2.98^{+0.39}_{-0.38}$ mJ/m^2
Ultraflex Free Surface (011 interface) (γ)	$41.89^{+14.56}_{-3.85}$ mJ/m^2	$43.67^{+6.37}_{-4.07}$ mJ/m^2	$1.22^{+1.7}_{-1.75}$ mJ/m^2	$-2.12^{+0.46}_{-0.43}$ mJ/m^2
TATB(001)-Ultraflex (Γ)	$32.08^{+5.51}_{-6.66}$ mJ/m^2	$8.1^{+3.85}_{-3.99}$ mJ/m^2	$16.59^{+5.67}_{-5.95}$ mJ/m^2	$7.58^{+3.34}_{-3.42}$ mJ/m^2
TATB(011)-Ultraflex (Γ)	$74.07^{+5.96}_{-6.99}$ mJ/m^2	$33.25^{+5.27}_{-5.34}$ mJ/m^2	$4.35^{+5.53}_{-6.02}$ mJ/m^2	$36.47^{+6.78}_{-6.23}$ mJ/m^2
TATB(001)-TATB(011) (Γ)	$102.98^{+2.07}_{-2.07}$ mJ/m^2	$48.52^{+1.57}_{-2.43}$ mJ/m^2	$77.09^{+1.58}_{-1.75}$ mJ/m^2	$-22.04^{+1.51}_{-1.72}$ mJ/m^2
TATB(001)-Ultraflex (Γ')	$-107.68^{+5.94}_{-7.08}$ mJ/m^2	$-114.21^{+4.17}_{-4.26}$ mJ/m^2	$7.29^{+5.97}_{-6.24}$ mJ/m^2	$-0.55^{+3.48}_{-3.55}$ mJ/m^2
TATB(011)-Ultraflex (Γ')	$-125.47^{+15.79}_{-8.04}$ mJ/m^2	$-114.98^{+8.3}_{-6.77}$ mJ/m^2	$-7.6^{+5.88}_{-6.37}$ mJ/m^2	$-3.86^{+6.86}_{-6.32}$ mJ/m^2
TATB(001)-TATB(011) (Γ')	$-161.73^{+2.71}_{-2.57}$ mJ/m^2	$-143.82^{+1.86}_{-2.66}$ mJ/m^2	$58.21^{+2.15}_{-2.35}$ mJ/m^2	$-75.6^{+1.99}_{-2.15}$ mJ/m^2

Acknowledgement

The authors thank Larry Fried, Emily Hoff, Amitesh Maiti, Paul Martinez, and Jeremy Scher for many useful discussions.

This work was supported by the Laboratory Directed Research and Development Program at Lawrence Livermore National Laboratory; project 24-SI-004 with Kyle Sullivan as P.I. This work was performed under the auspices of the U.S. Department of Energy by Lawrence Livermore National Laboratory under Contract DE-AC52-07NA27344. Approved for unlimited release: LLNL-JRNL-2019872.

References

(S1) Gennes, P. G. d. *Scaling concepts in polymer physics*; Cornell University Press: Ithaca, N.Y, 1979.

(S2) Brünger, A.; Brooks III, C. L.; Karplus, M. Stochastic boundary conditions for molecular dynamics simulations of ST2 water. *Chemical physics letters* **1984**, *105*, 495–500.
